# Supplementary material for: Arf1-mediated lipid metabolism sustains cancer cells and its ablation induces anti-tumor immune responses in mice
Source: Nat Commun. 2020 Jan 10;11:220. doi: 10.1038/s41467-019-14046-9 (PMC6954189; doi:10.1038/s41467-019-14046-9)
Supplement: Supplementary file 1 — Supplementary Information [file 41467_2019_14046_MOESM1_ESM.pdf]

Arf1-mediated Lipid Metabolism Sustains Cancer Cells and Its ablation Induces Anti-tumor Immune Responses in Mice

Wang G et al.

Supplementary Information

Supplementary Figures

Supplementary Figure 1

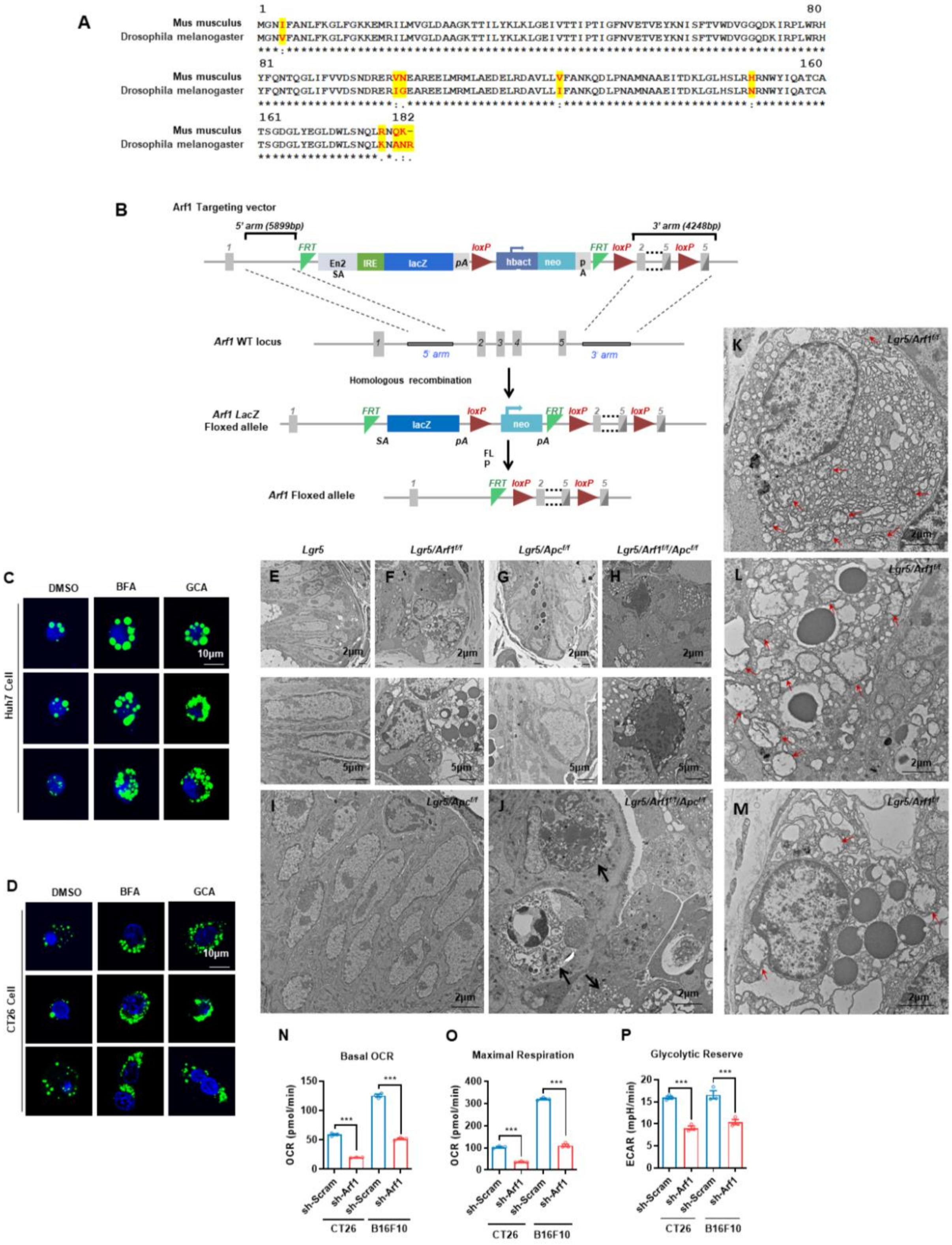

Supplementary Figure 1. Arf1 ablation induced lipid droplet accumulation and mitochondrial defects in mouse intestine. (A) Alignment of the amino acid sequences of mouse and *Drosophila* Arf1. (B) Diagram depicting the procedure used to generate Arf1 conditional knockout mice. (C) Huh-7 cells were treated with the indicated compounds and stained with a lipid droplet marker (green) and Hoechst (blue). Scale bars, 10 μm. (D) CT26 cells were treated with the indicated compounds and stained with a lipid droplet marker (green) and Hoechst (blue). Scale bars, 10 μm. (E-M) Electron microscopy sections of mouse intestinal crypts with the indicated genotypes. Arf1 knockdown mice showed cells with abundant broken and swollen mitochondria (red arrows), poor cristae, numerous vacuoles, degeneration, and necrosis (black arrows). (N-P) Oxygen consumption and glycolytic reserve rates were significantly lower in Arf1-knockdown (sh-Arf1) CT26 and B16-F10 cells compared with Scram (control) knockdown cells (n=3 wells cell each group; *$p<0.05$, **$p<0.01$ and ***$p<0.001$, t-test; repeat two independent experiments). Data are shown as the mean +/- SEM. Scale bars, 2 μm and 5 μm.

Supplementary Figure 2

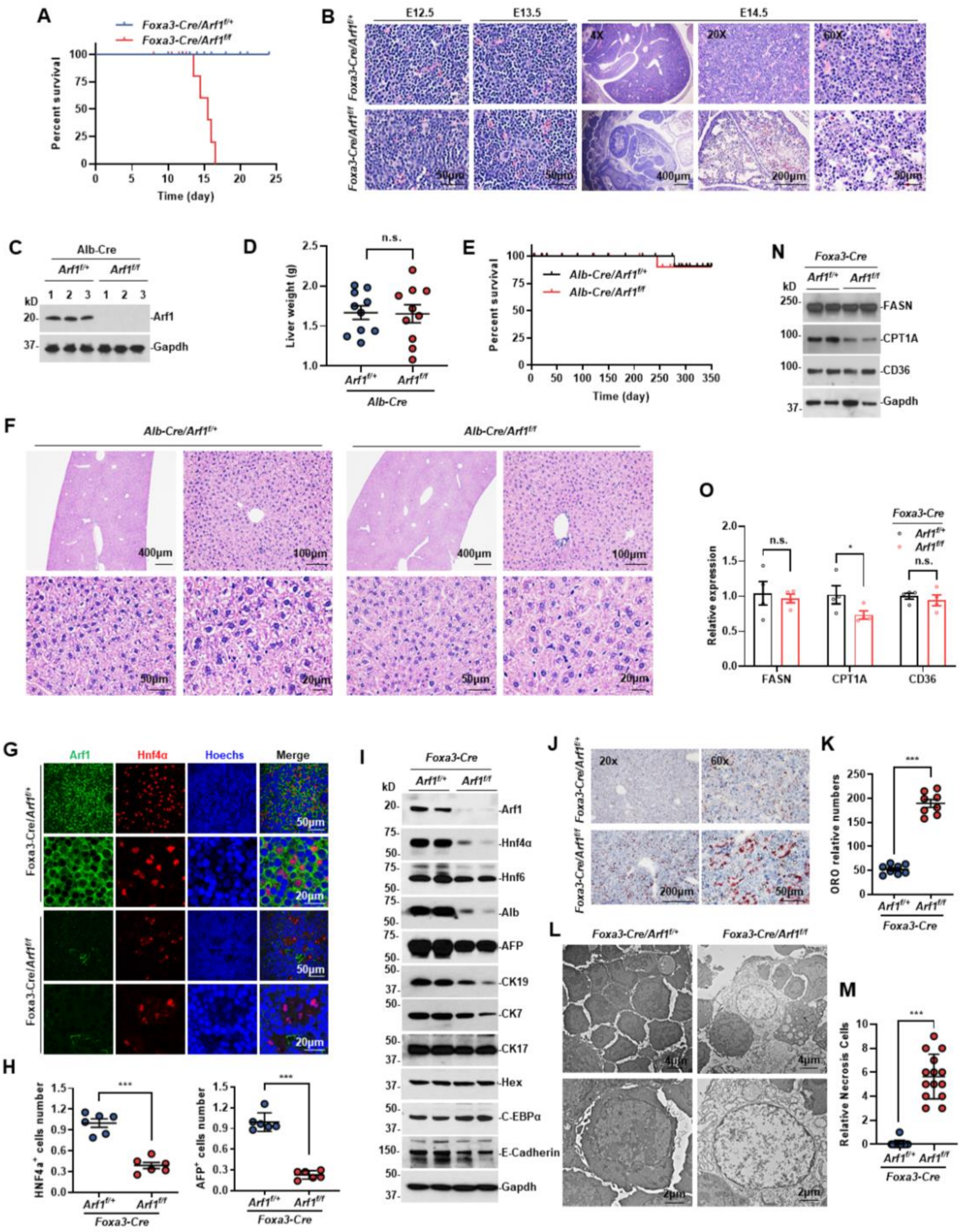

Supplementary Figure 2. *Arf1* knockdown in mouse embryos results in hepatoblast defects. (A) Survival curves of mice with the indicated genotypes (n=15 mice each group; $*p<0.05$, $**p<0.01$ and $***p<0.001$, t-test; repeat three independent experiments). (B) Hematoxylin and eosin (H&E) stained

control (*Foxa3-Cre/Arf1*$_{f/+}$) or *Arf1* knockdown (*Foxa3-Cre/Arf1*$_{f/f}$) mouse livers. Note that there was more cell death in the *Arf1* knockdown liver. (C) *Alb-Cre* effectively depleted *Arf1*. (D) Liver weight of the indicated genotypes. (E) Survival curves of the indicated genotypes (n=10 mice each group; *p<0.05, **p<0.01 and ***p<0.001, t-test; pooled two independent experiments). (F) H&E stained control (*Alb-Cre/Arf1*$_{f/+}$) or *Arf1* knockdown (*Alb-Cre/Arf1*$_{f/f}$) mouse livers. Note that there was no difference between the control and *Arf1* knockdown livers. (G) Immunofluorescence staining of Arf1 (green), Hnf4α (red), and Hoechst (blue) in *Arf1*-deleted (*Foxa3-Cre/Arf1*$_{f/f}$) and heterozygous control (*Foxa3-Cre/Arf1*$_{f/+}$) embryos. (H) Quantification of Hnf4α and AFP expression in *Foxa3-Cre/Arf1*$_{f/f}$ and *Foxa3-Cre/Arf1*$_{f/+}$ embryos. (I) Western blots of the indicated proteins in *Foxa3-Cre/Arf1*$_{f/f}$ (KO) and *Foxa3-Cre/Arf1*$_{f/+}$ (Control) embryos. (J) lipid droplet (red) accumulation in *Foxa3-Cre/Arf1*$_{f/f}$ (KO) and *Foxa3-Cre/Arf1*$_{f/+}$ (Control) embryos. (K) Quantification of lipid droplets in *Foxa3-Cre/Arf1*$_{f/f}$ (KO) and *Foxa3-Cre/Arf1*$_{f/+}$ (Control) embryos (n=5 mice each group; *p<0.05, **p<0.01 and ***p<0.001, t-test; repeat two independent experiments). (L) EM images of liver sections showing the necrotic death of hepatoblasts in the *Foxa3-Cre/Arf1*$_{f/f}$ (KO) but not the *Foxa3-Cre/Arf1*$_{f/+}$ mouse (Control). (M) Quantification of the relative necrotic cells in *Foxa3-Cre/Arf1*$_{f/f}$ (KO) and *Foxa3-Cre/Arf1*$_{f/+}$ (Control) embryos (n=15 mice each group; *p<0.05, **p<0.01 and ***p<0.001, t-test; repeat three independent experiments). (N-O) Western blot (N) and qRT-PCR (O) of indicated protein and genes (n=5 mice each group, repeat three independent experiments). Data are shown as the mean +/- SEM. ***p<0.001 by Student's t test. Scale bars are as indicated.

Supplementary Figure 3

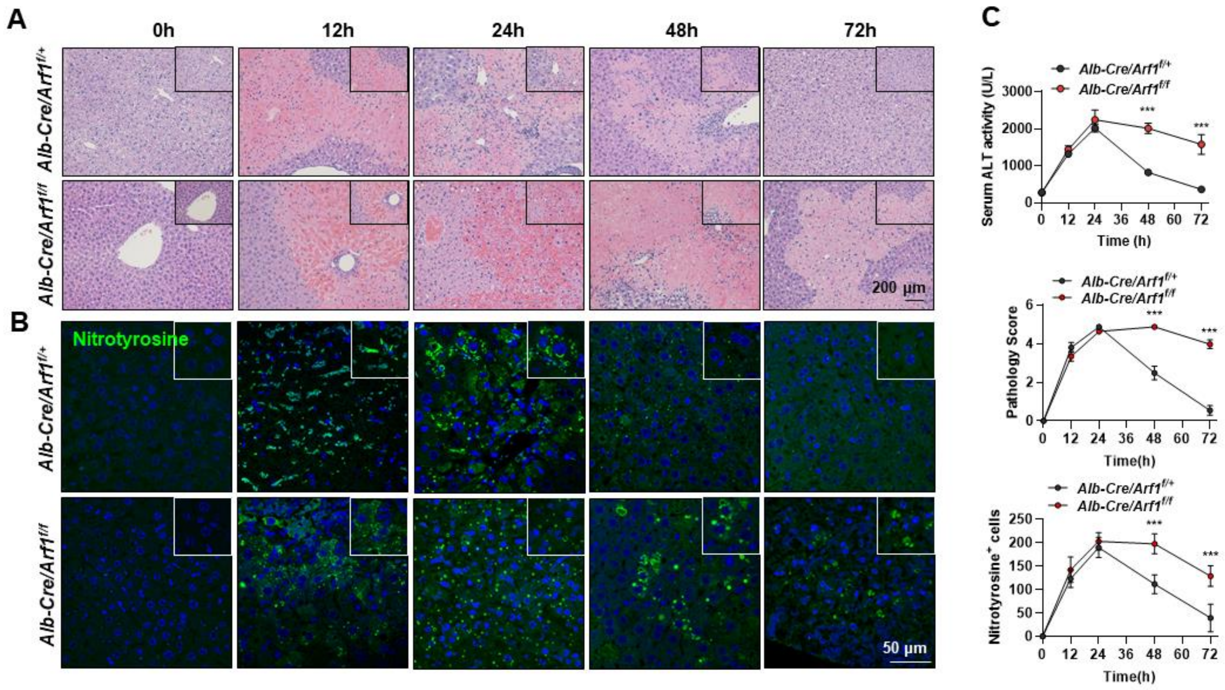

Supplementary Figure 3. *Arf1* knockdown in mouse liver results in delay hepatocytes regeneration. (A) H&E stained different time points of control (*Alb-Cre/Arf1*$_{f/+}$) or *Arf1* knockdown (*Alb-Cre/Arf1*$_{f/f}$) mouse livers after treatment with APAP. (B) Nitrotyrosine stained of control (*Alb-Cre/Arf1*$_{f/+}$) or *Arf1* knockdown (*Alb-Cre/Arf1*$_{f/f}$) mouse livers after treatment with APAP (300 mg/kg). (C) ELISA of ALT level, quantification of pathology score and quantification of Nitrotyrosine positive cells in the control (*Alb-Cre/Arf1*$_{f/+}$) or *Arf1* knockdown (*Alb-Cre/Arf1*$_{f/f}$) mouse livers after treatment with APAP. Scale bar as 200 μm (A) and 50 μm (B). n=5 mice each group; *p<0.05, **p<0.01 and ***p<0.001 by Student's t test; single experiments. Data are shown as the mean +/- SEM.

Supplementary Figure 4

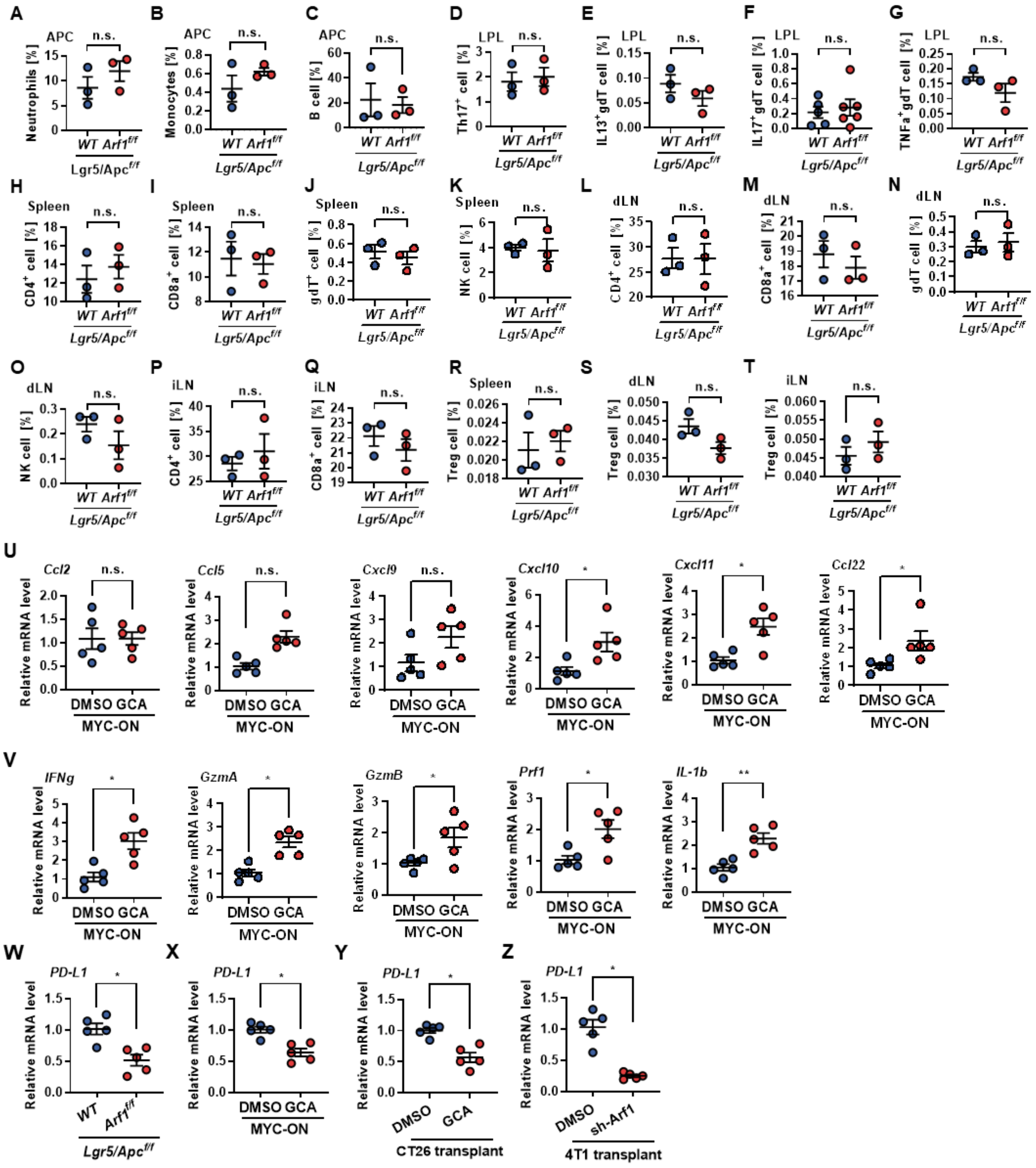

Supplementary Figure 4. Arf1 ablation induces the infiltration and activation of immune cells in intestine and liver tumor mice. (A-C) Flow cytometric analysis of gut APCs: neutrophils (A), monocytes (B), and B cells (C). (D-G) Flow cytometric analysis of the immune cells of LPLs: Th17 cells (D), IL-13$^+$ γδ T cells (E), IL-17$^+$ γδ T cells (F), and TNFα$^+$ γδ T cells (G). (H-K) Flow cytometric analysis of immune cells of the spleen: CD4$^+$ T cells (H), CD8α$^+$ T cells (I), γδ T cells (J), and NK cells

(K). (L-O) Flow cytometric analysis of the immune cells of dNL lymph nodes: $CD4^+$ T cells (L), $CD8\alpha^+$ T cells (M), γδ T cells (N), and NK cells (O). (P-Q) Flow cytometric analysis of the immune cells of inguinal lymph nodes: $CD4^+$ T cells (P) and $CD8\alpha^+$ T cells (Q). (R-T) Flow cytometric analysis of Treg cells of the spleen (R), dNL lymph node (S), and inguinal lymph node (T). (U) Relative gene expression of the indicated chemokines from the liver of MYC-ON liver tumor mice treated with the Arf1 inhibitor GCA or DMSO. (V) Relative gene expression of the indicated cytokines and granzymes from the liver of *MYC-ON* liver tumor mice treated with the Arf1 inhibitor GCA or DMSO. (W-Z) Relative PD-L1 expression in the intestine of *Lgr5/Apc*$^{f/f}$ or *Lgr5/Apc* $^{f/f}$*/Arf1*$^{f/f}$ mice (W), in the tumors of *MYC-ON* liver tumor mice (X), mice with CT26-transplanted syngeneic tumors (Y), or mice with 4T1-transplanted syngeneic tumors (Z). A-E group, n=3 mice; F, n=5 mice; G-T, n=3 mice; U-Z, n=5 mice per group; *$p<0.05$, **$p<0.01$, ***$p<0.01$, t-test; repeat two independent experiments. Data are shown as the mean +/- SEM.

Supplementary Figure 5

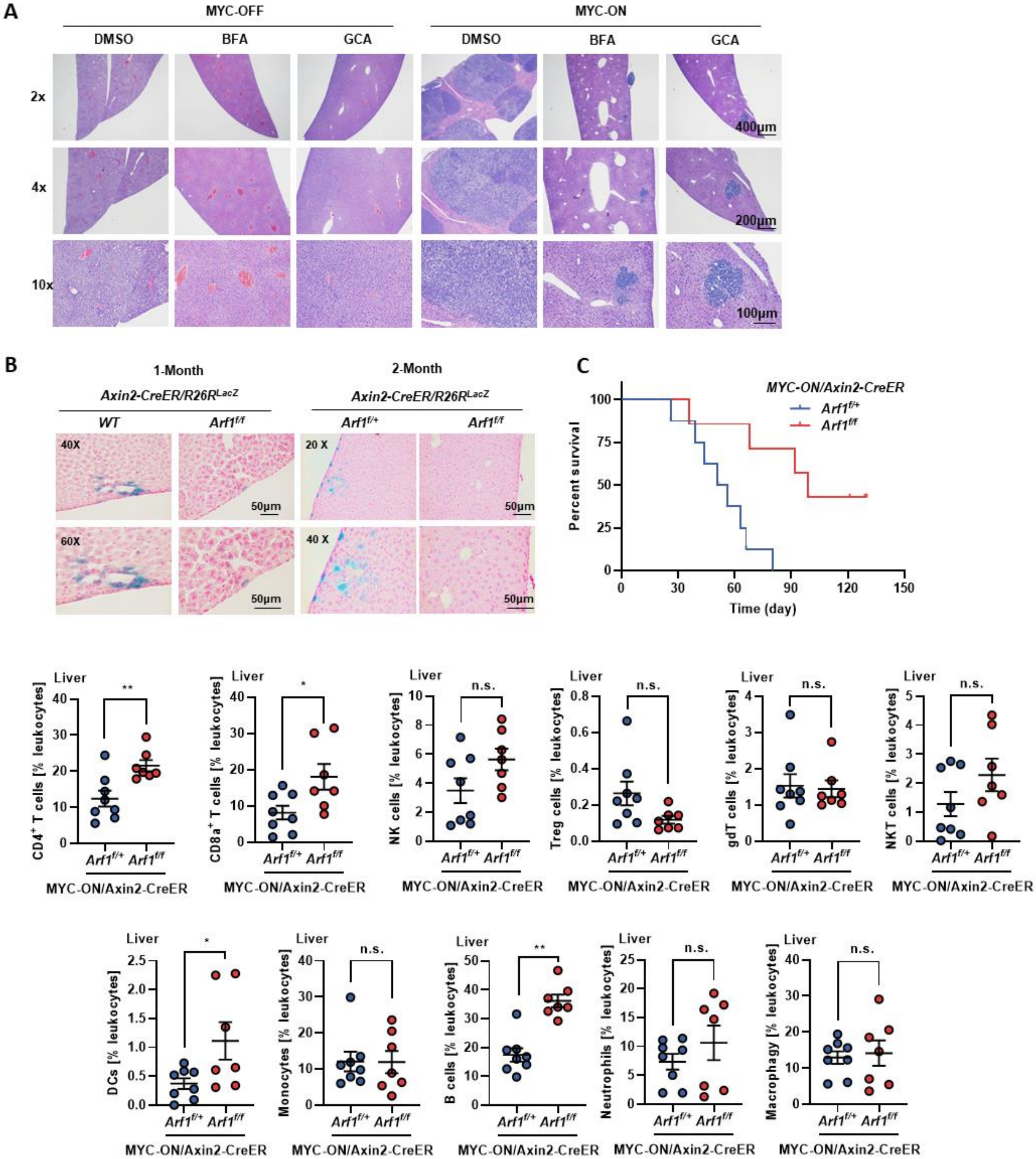

Supplementary Figure 5. Arf1 inhibition induces anti-tumor immune responses in MYC-ON liver tumor mice. (A) Representative H&E staining of MYC-OFF or MYC-ON mice treated with DMSO, GCA, or BFA. (B) LacZ-stained sections of the liver from an *Axin2-CreER-cre/Rosa26R* or an *Axin2-CreER-Cre/Rosa26R/Arf1*f/f mouse that was treated with a single intraperitoneal injection of tamoxifen to

activate the stem-cell-specific Cre and facilitate the loss of Arf1. Note that the LacZ-positive stem cells were effectively eliminated in the *Arf1*-knockdown mice. (C) Survival curves of *MYC-ON/Axin2-CreER/Arf1*$_{f/+}$ and *MYC-ON/Axin2-CreER/Arf1*$_{f/f}$ mice (n=10 mice each group; *p<0.05, **p<0.01, ***p<0.01, t-test; pooled two independent experiments). (D) Flow cytometric analysis of immune cells in the liver of mice with the indicated genotypes (n=8 mice each group; *p<0.05, **p<0.01, ***p<0.01, t-test; pooled two independent experiments). Data are shown as the mean +/- SEM. Scale bars are as indicated.

Supplementary Figure 6

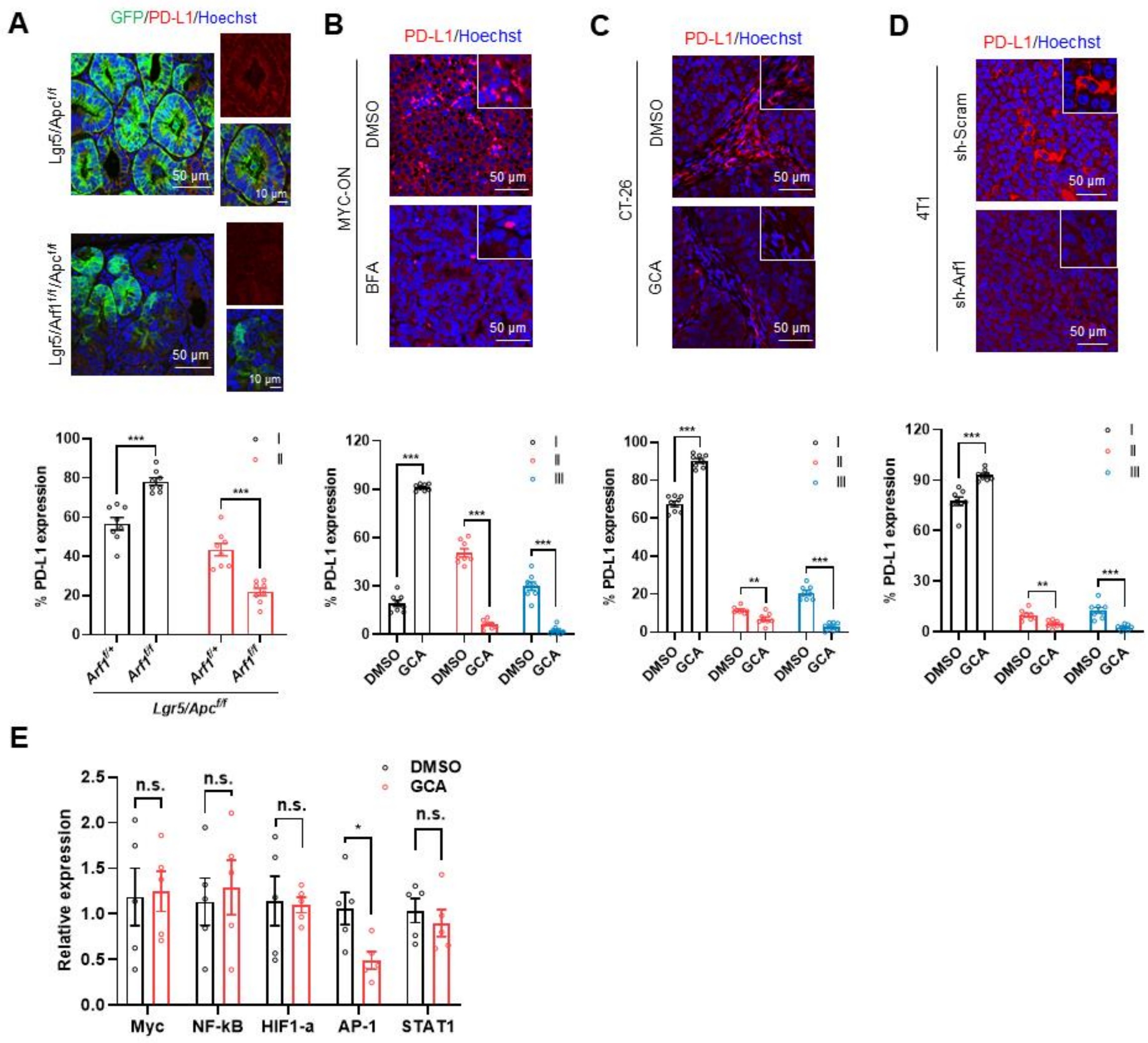

Supplementary Figure 6. Immunofluorescence stained of PD-L1 in the difference tumor models. (A-D) IF stained and quantification of PD-L1 level in the intestine of Lgr5/Apc and Lgr5/Arf1/Apc mice (A), liver of MYC-ON mice (B), transplant CT26 tumor (C) and transplant 4T1 tumor (D) (n=8 area each group; *p<0.05, **p<0.01, ***p<0.01, t-test; repeat three independent experiments). (E) qRT-PCR amplifies the transcription factors in the liver of DMSO or GCA treated MYC-ON mice (n=5 mice each group; *p<0.05, **p<0.01, ***p<0.01, t-test; repeat three independent experiments). Data are shown as the mean +/- SEM. Scale bars are as indicated.

Supplementary Figure 7

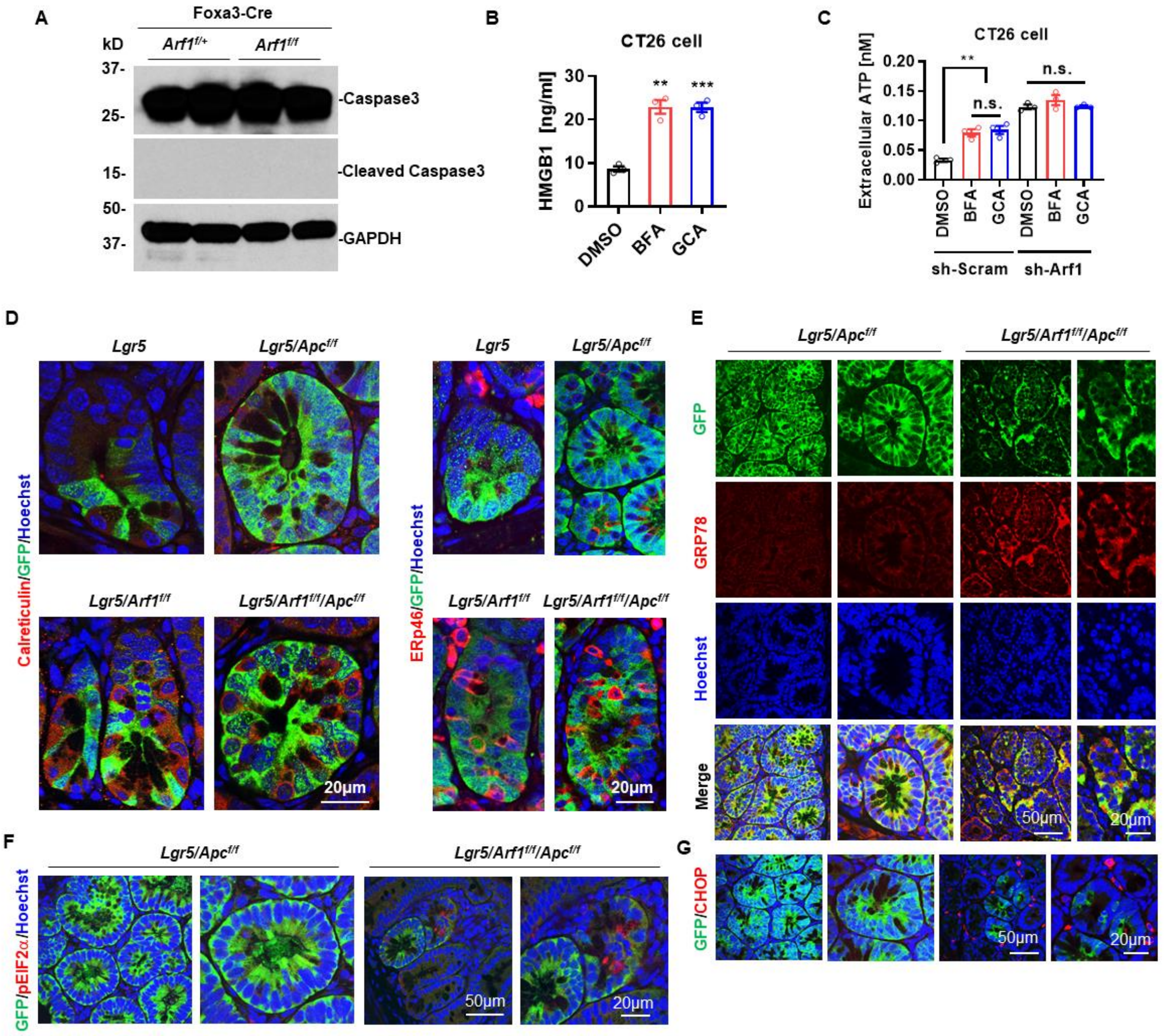

Supplementary Figure 7. *Arf1* knockdown triggers ER stress and induces DAMPs in Lgr5/Apc mice. (A) Western blot of Caspase3 and cleaved caspase 3 in the *Foxa3-Cre/Arf1f/+* and *Foxa3-Cre/Arf1f/f* mice. (B) Direct treatment of CT26 cells with the Arf1 inhibitor BFA or GCA induced the expression of HMGB1 (n=3 well cells per group; **p<0.01, ***p<0.001 t-test; repeat three independent experiments). (C) Treatment of CT26 cells with the Arf1 inhibitor BFA or GCA induced ATP secretion in control but not in Arf1 (sh-Arf1) knockdown cells (n=3 well cells per group; **p<0.01, ***p<0.001 t-test; repeat three independent experiments). (D) Calr and ERp46 were significantly induced in the intestine of Arf1-knockdown mice. (E-F) The ER stress markers GRP78/BiP, peIF2α, and CHOP were stained in the intestine of *Arf1*-knockdown mice. Scale bars are as indicated. Data are shown as the mean +/- SEM. **p<0.01, ***p<0.001 by Student's t test.

Supplementary Figure 8

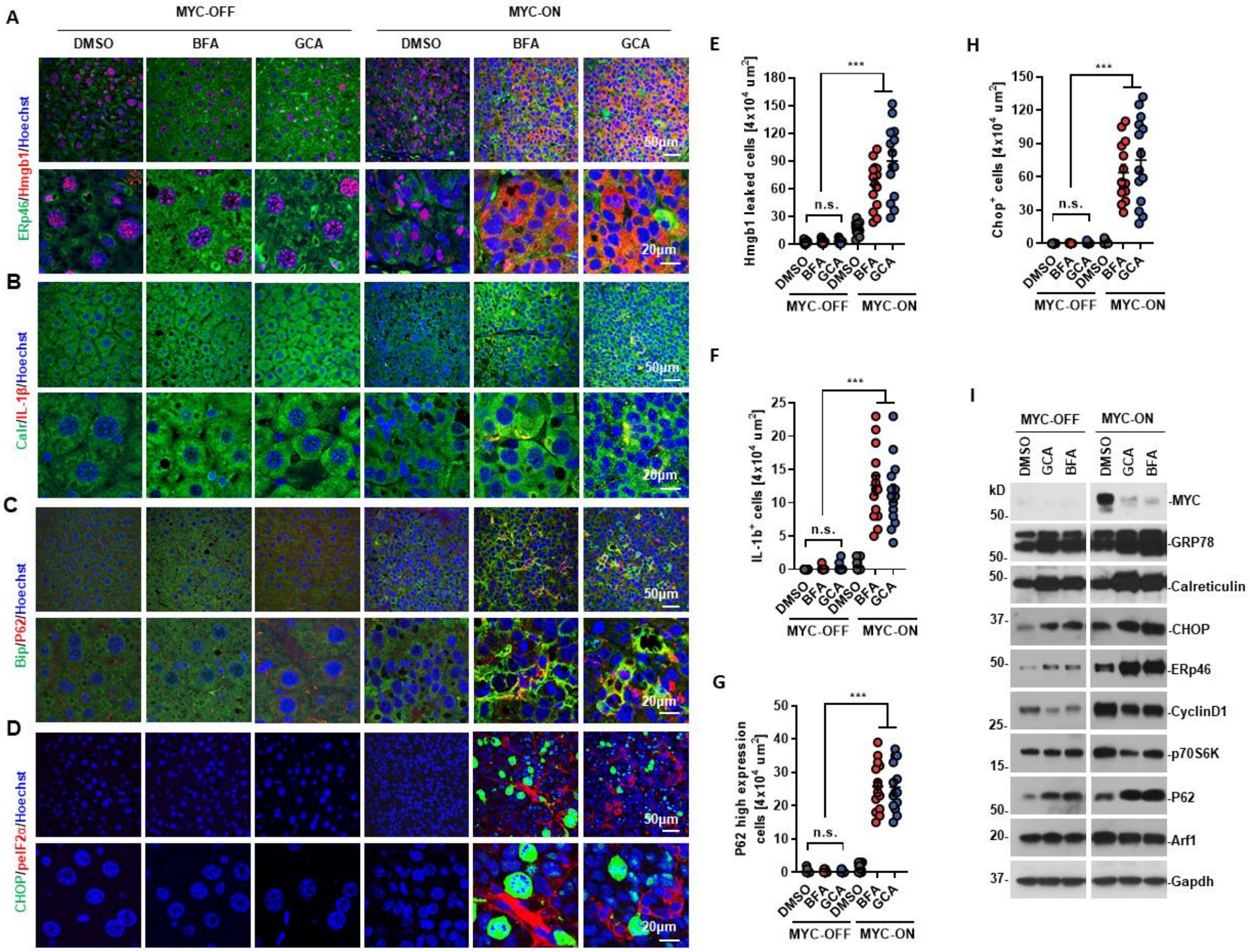

Supplementary Figure 8. Arf1 Inhibition triggers ER stress and induces DAMPs in liver tumor mice. *MYC-OFF* or *MYC-ON* liver tumor mice were treated with the indicated reagents. The ER stress markers (BiP, peIF2α, and CHOP), DAMPs (Calr, ERp46, and HMGB1), IL-1β, and p62 were significantly increased in the liver of mice treated with the Arf1 inhibitor GCA or BFA compared with control mice treated with DMSO. (A-D) are tumor sections stained for the indicated markers. (E-H) are quantifications of the indicated markers (n=15 areas each group; *p<0.05, ***p<0.001, t-test; repeat three independent experiments). (I) western blot of the indicated proteins. Data are shown as the mean +/- SEM. ***p<0.001 by Student's t test. Scale bars are as indicated.

Supplementary Figure 9

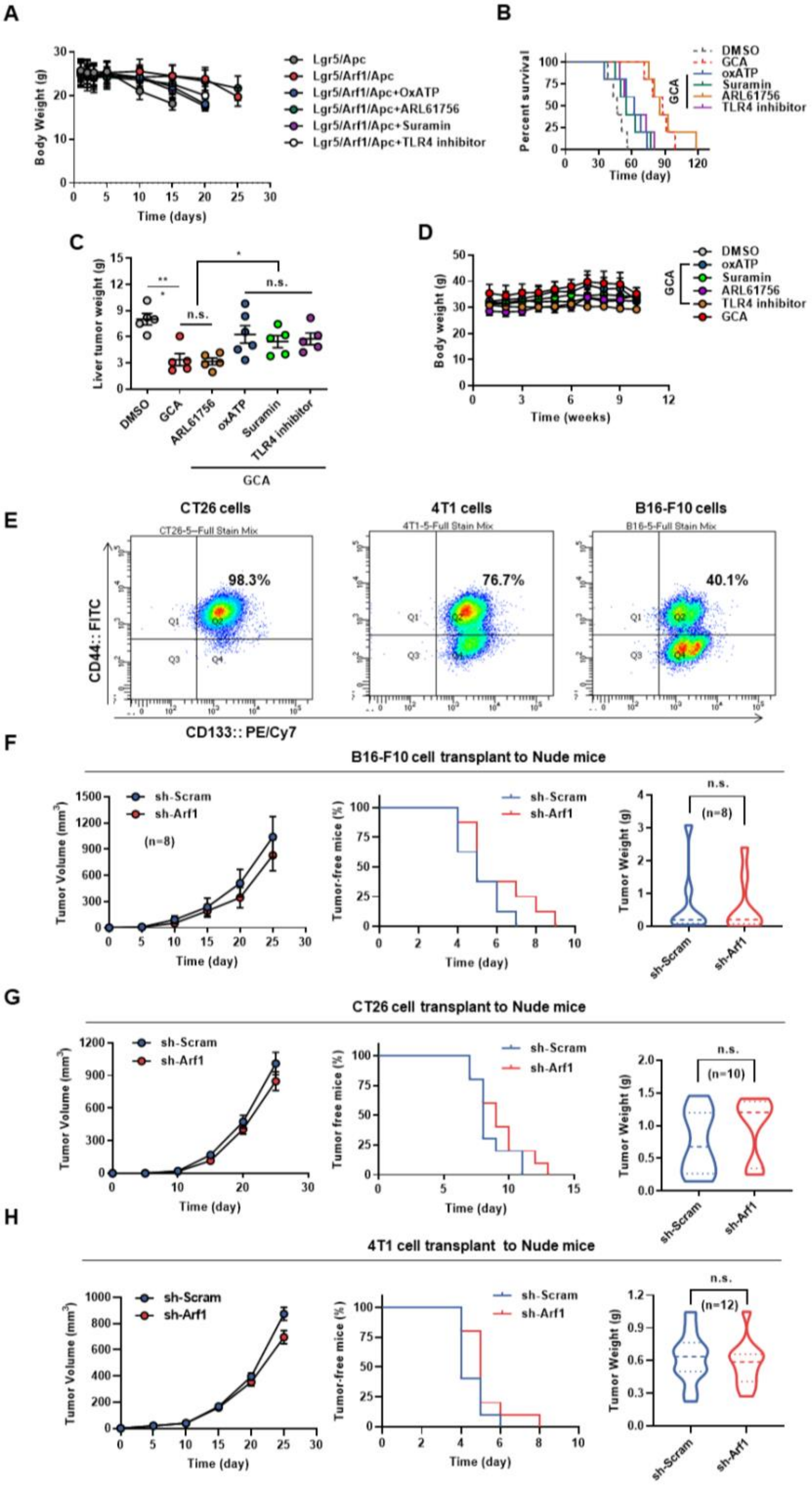

Supplementary Figure 9. Arf1 knockdown does not have anti-tumor activity in immune-deficient mice. (A) Body weight curves of *Lgr5/Arf1/Apc* mice treated with the indicated reagents. (B) Survival

curves of MYC-ON mice treated with the indicated reagents (n=5 mice each group; *$p<0.05$, ***$p<0.001$ t-test; repeated three independent experiments). (C) Liver tumor weight of MYC-ON mice treated with the indicated reagents. (D) Body weight curves of MYC-ON mice treated with the indicated reagents (n=5 mice each group, repeated three independent experiments). (E) Flow cytometry analysis the CD133+ and CD44+ stem cells in the CT26, B16F10 and 4T1 cells. (F) sh-Scram- or sh-Arf1-treated B16-F10 cells were injected into Athymic mice (n=8 mice each group, pooled two independent experiments). (G) sh-Scram- or sh-Arf1-treated CT26 cells were injected into Athymic mice (n=10 mice each group, pooled two independent experiments). (H) sh-Scram- or sh-Arf1-treated 4T1 cells were injected into Athymic mice. The tumor volumes, tumor-free mice, and tumor weights were similar in all the control and Arf1-ablated mice. (n=12 mice each group, pooled three independent experiments). Data are shown as the mean +/- SEM.

Supplementary Figure 10

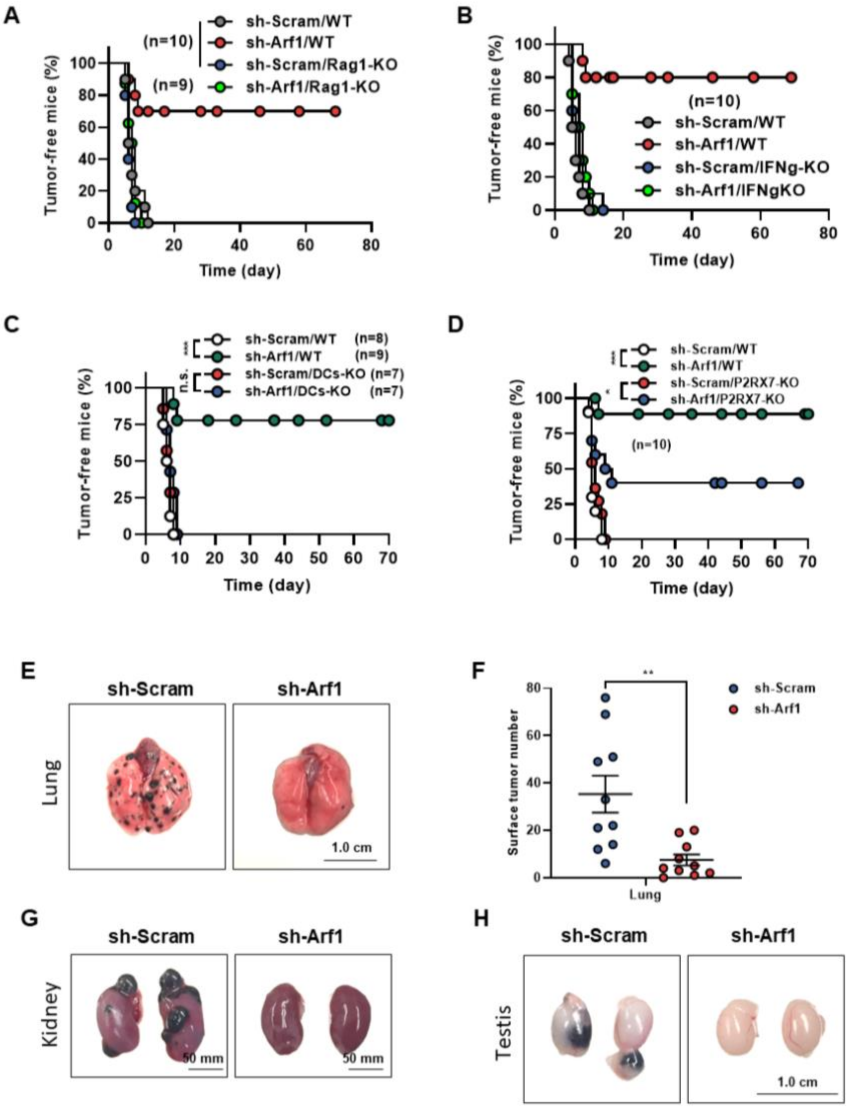

Supplementary Figure 10. Arf1 ablation induced anti-tumor immune response and blocks tumor metastasis. (A) Tumor-free curves of wild-type or *Rag1-KO* C57BL/6J mice receiving B16-F10 cells treated with the indicated sh-RNAs. (B) Tumor-free curves of wild-type or *IFNγ-KO* BALB/c mice receiving CT26 cells treated with the indicated sh-RNAs. (C) Tumor-free curves of wild-type or *DCs-KO* C57BL/6J mice receiving B16-F10 cells treated with the indicated sh-RNAs. (D) Tumor-free curves of wild-type or P2RX7-KO C57BL/6J mice receiving B16-F10 cells treated with the indicated sh-RNAs. (E-H) Arf1 knockdown in tumor cells prevents tumor metastasis. B16-F10 cells that were transfected with either sh-Arf1 (sh-Arf1-B16) or sh-Scram (sh-Scram-B16) were injected into tail vain of C57BL/6J mice. The tumors in sh-Scram-B16 mice metastasized to lung (E and F), kidney (G) and testis (H) after 15 days

of injection, while the tumors in sh-Arf1-B16 mice had negligible tumor metastasis. (A, WT, n=10, Rag1-KO, n=9; B and D, n=10; C, sh-Scram/WT, n=8, sh-Arf1/WT, n=9, sh-Scram/DCs-KO and sh-Arf1/DCs-KO, n=7 mice each group; *$p<0.05$, **$p<0.01$, ***$p<0.001$, t-test; pooled two independent experiments. Data are shown as the mean +/- SEM. **$p<0.01$ by Student's t test. Scale bars are as indicated.

Supplementary Figure 11

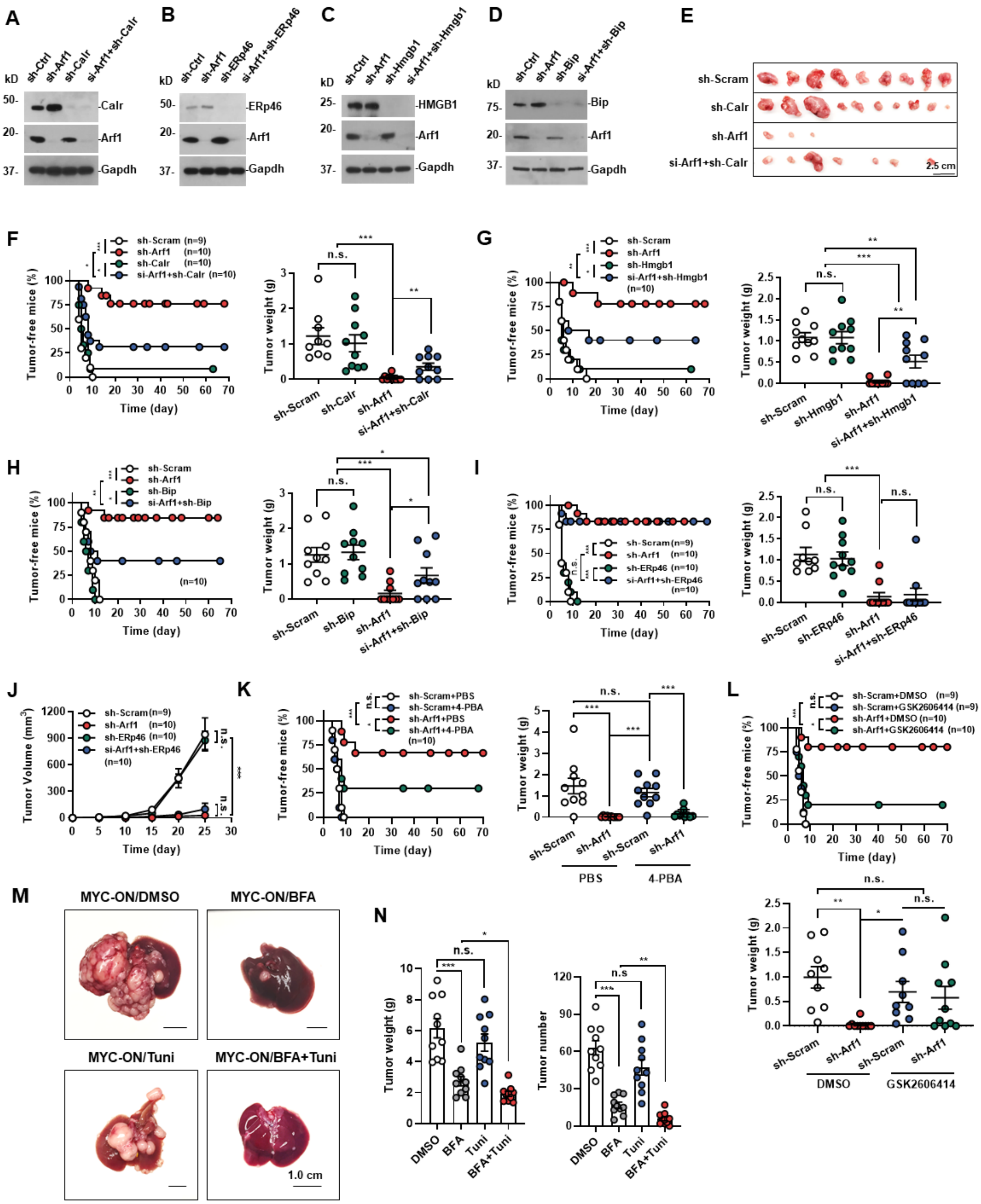

Supplementary Figure 11. Arf1-KO in CT26 cells induces anti-tumor immune responses through DAMPs. (A-D) Specific small interfering RNAs (sh-RNAs) effectively knocked down the indicated

genes. (E) Tumor sizes in BALB/c mice receiving CT26 cells that were transfected with the indicated shRNAs. Scale bars are as indicated. (F-L) Tumor volume curves, tumor-free curves, and tumor weights of BALB/c mice receiving CT26 cells treated with the indicated sh-RNAs (F, J, sh-Scram, n=9, other group, n=10; G, H, K, n=10 mice each group; I, sh-Scram, n=9, other group, n=10; L, sh-Scram/DMSO and sh-Scram/GSK2606414, n=9, sh-Arf1/DMSO and sh-Arf1/GSK2606414, n=10 mice per group; *$p<0.05$, **$p<0.01$, ***$p<0.001$, t-test; single experiments). (M) Surface tumor of MYC-ON mice treated with BFA, Tunicamycin, or BFA+Tunicamycin. (N) Liver weight and liver tumor number in the (M) (n=10 mice each group; *$p<0.05$, **$p<0.01$, ***$p<0.001$, t-test; single experiments). Data are shown as the mean +/- SEM. *$p<0.05$, **$p<0.01$, ***$p<0.001$ by Student's t test.

Supplementary Figure 12

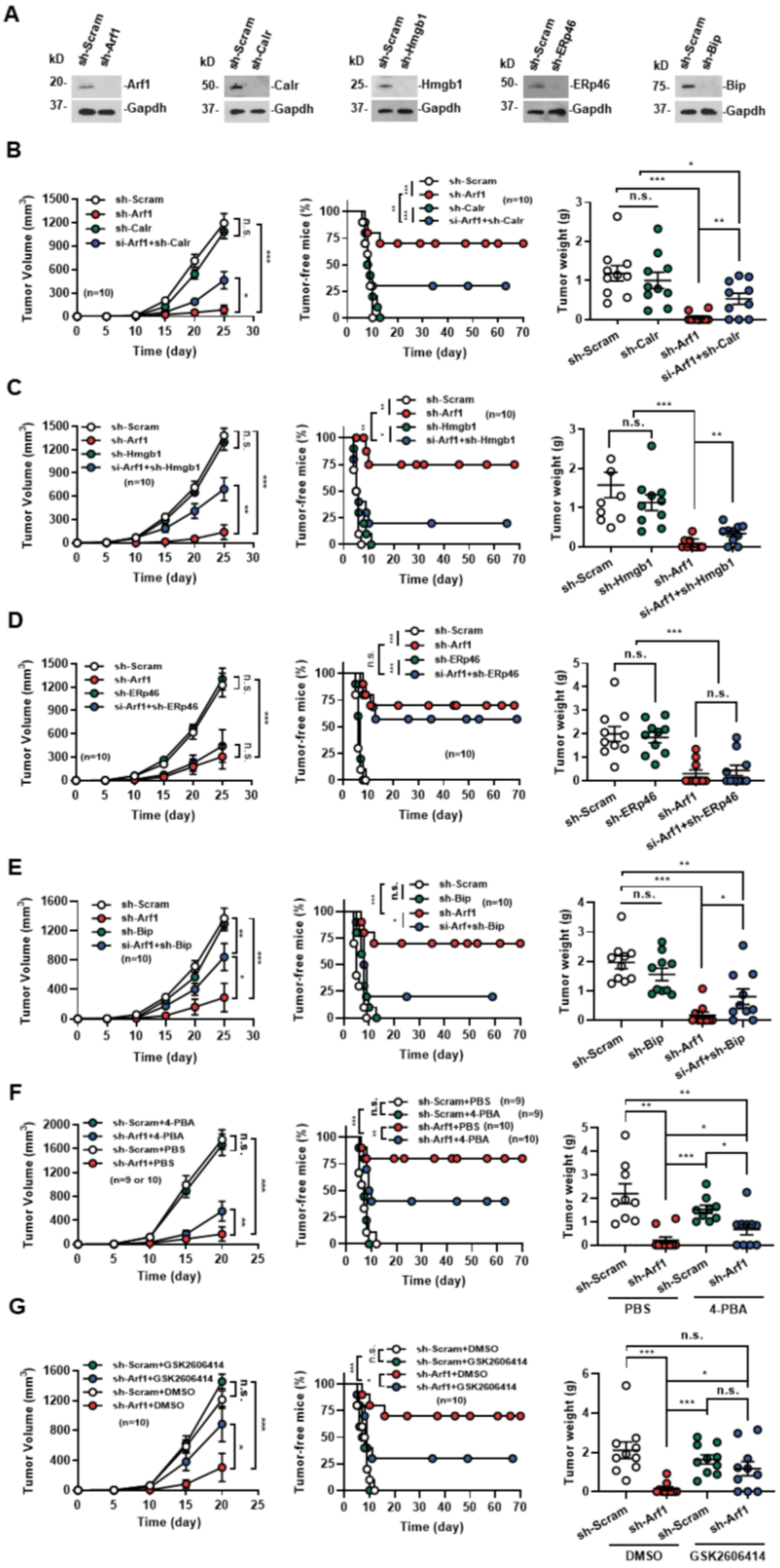

Supplementary Figure 12. Arf1 ablation induces anti-tumor immune responses through DAMPs. (A) Specific small interfering RNAs (sh-RNAs) effectively knocked down the indicated genes. (B-G) Tumor volume curves, tumor-free curves, and tumor weights of C57BL/6J mice receiving murine melanoma B16-F10 cells treated with the indicated sh-RNAs or inhibitors (B, C, D, E and G, n=10 mice each group; F, sh-Scram/PBS and sh-Scram/4-PBA, n=9, sh-Arf1/PBS and sh-Arf1/4-PBA, n=10 mice each group; *$p<0.05$, **$p<0.01$, ***$p<0.001$, t-test; polled two independent experiments). Data are shown as the mean +/- SEM.

Supplementary Figure 13

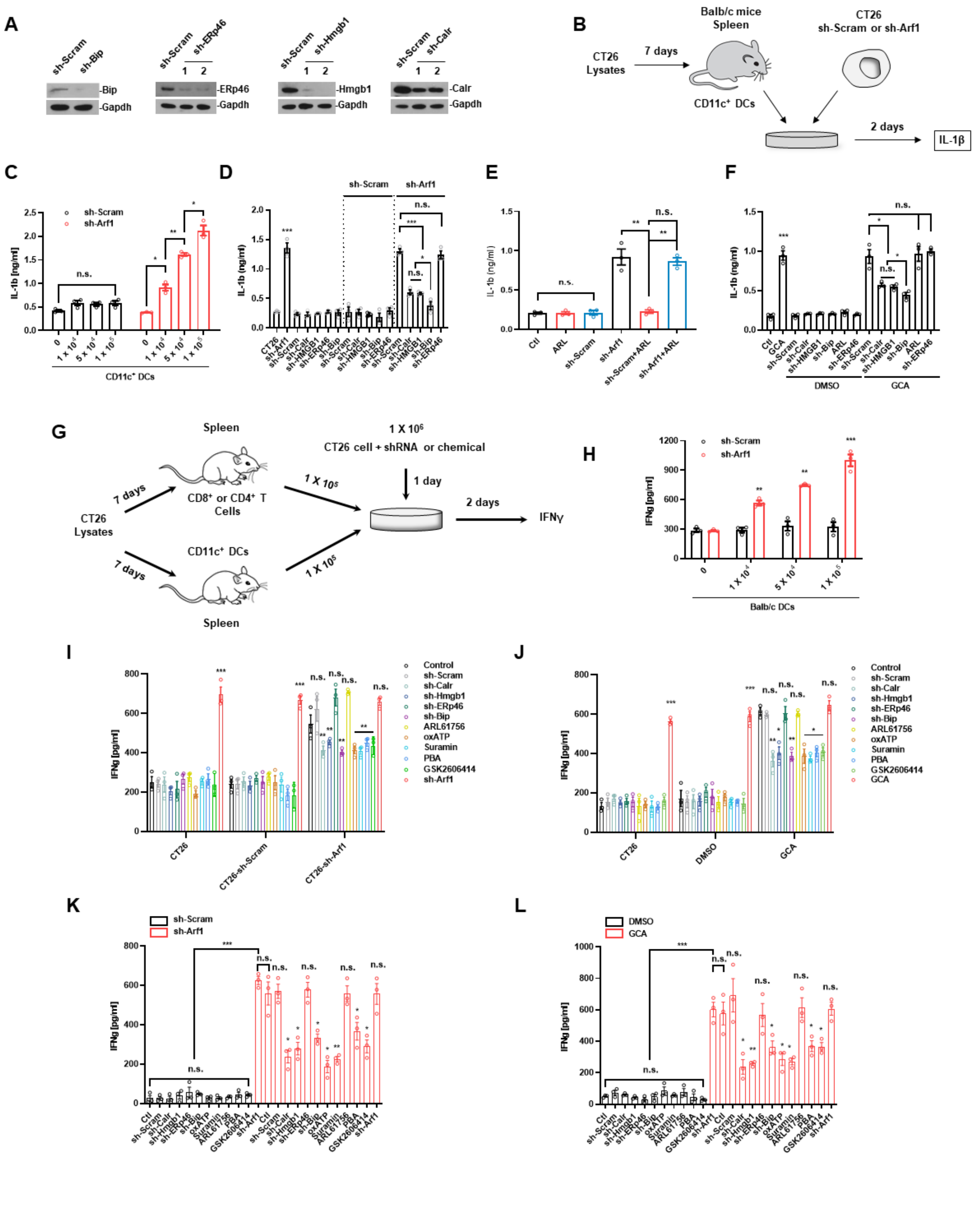

Supplementary Figure 13. Arf1 ablation induces the expression of IL-1β and IFNγ through DAMPs. (A) Specific small interfering RNAs (sh-RNAs) effectively knocked down the indicated genes in CT26 cells. (B) Experimental setup for the IL-1β experiments. After injecting CT26 cell lysates into BALB/c mice for 7 days, $CD11c^{+}$ DCs were isolated from the mice and cocultured with CT26 cells transfected with either sh-Scram or sh-Arf1. Two days later, IL-1β was measured in the harvested cells. (C-F) Quantification of IL-1β levels under the indicated treatment conditions. (G) Experimental setup for the INFγ experiments. After injecting CT26 cell lysates into BALB/c mice for 7 days, the $CD11c^{+}$ DCs and $CD4^{+}$ or $CD8^{+}$ T cells were isolated from the mice and cocultured with CT26 cells transfected with either sh-Scram or sh-Arf1. Two days later, INFγ was measured in the harvested cells. (H-L) Quantification of the INFγ levels under the indicated treatment conditions. (n=3 wells cell in each group; *$p<0.05$, **$p<0.01$, ***$p<0.001$, t-test; repeat three independent experiments). Data are shown as the mean +/- SEM.

Supplementary Figure 14

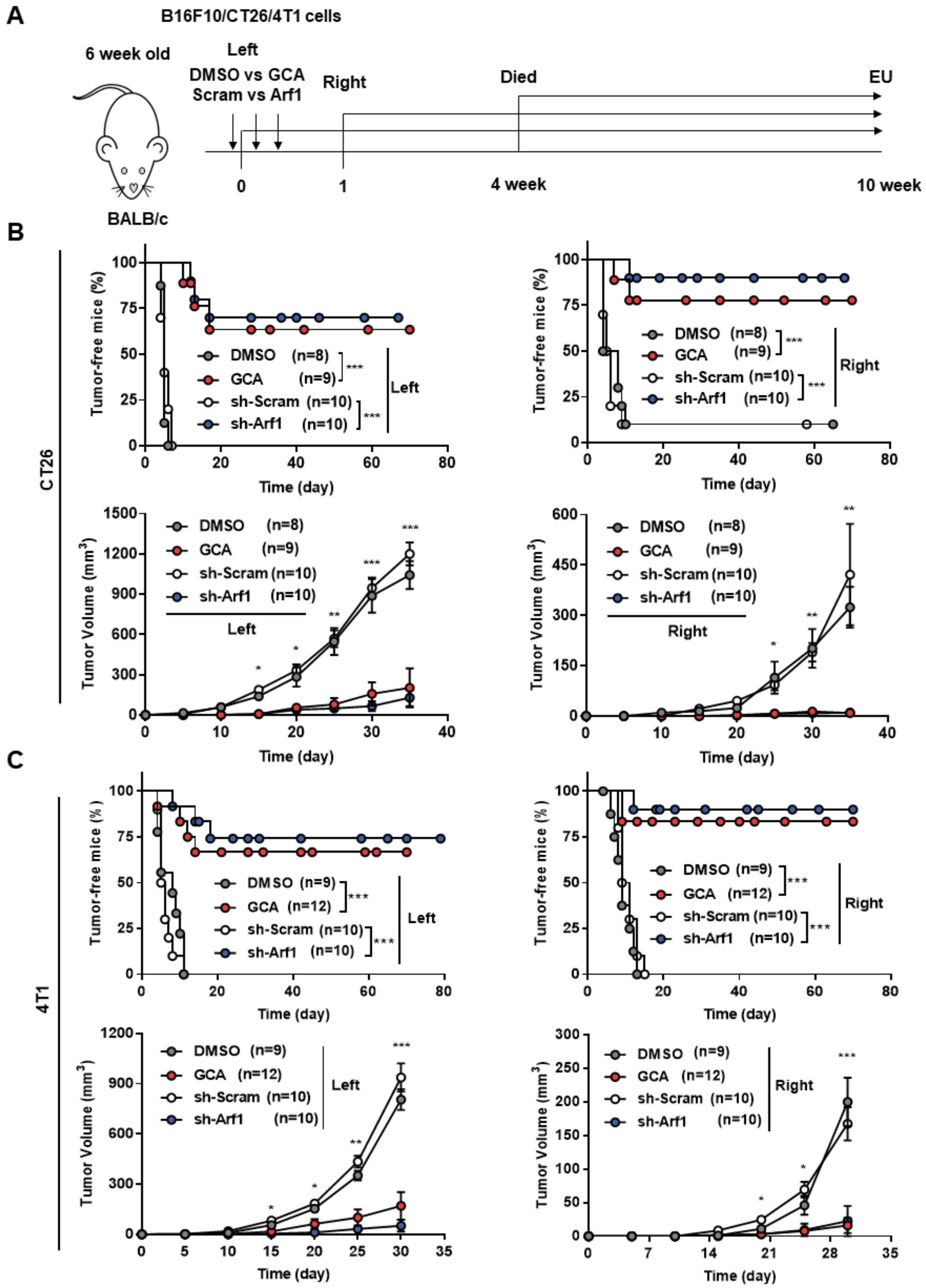

Supplementary Figure 14. Vaccination with Arf1-ablated cells protects animals from developing tumors. (A) Experimental setup. We injected CT26 or 4T1 cells that were transfected with sh-Arf1 or sh-Scram or treated with the Arf1 inhibitor GCA or DMSO into the left side of BALB/c mice, and then the

mice were rechallenged one week later with untreated CT26 or 4T1 cells on the right side. (B) Tumor-free and tumor volume curves of BALB/c mice injected with CT26 cells treated with the indicated reagents (DMSO, n=8; GCA, n=9; sh-Scram and sh-Arf1, n=10 mice each group; *p<0.05, **p<0.01, ***p<0.001, t-test; pooled two independent experiments). (C) Tumor-free and tumor volume curves of BALB/c mice injected with 4T1 cells treated with the indicated reagents (DMSO, n=9; GCA, n=12; sh-Scram and sh-Arf1, n=10 mice each group; *p<0.05, **p<0.01, ***p<0.001, t-test; pooled two independent experiments). Data are shown as the mean +/- SEM.

Supplementary Figure 15

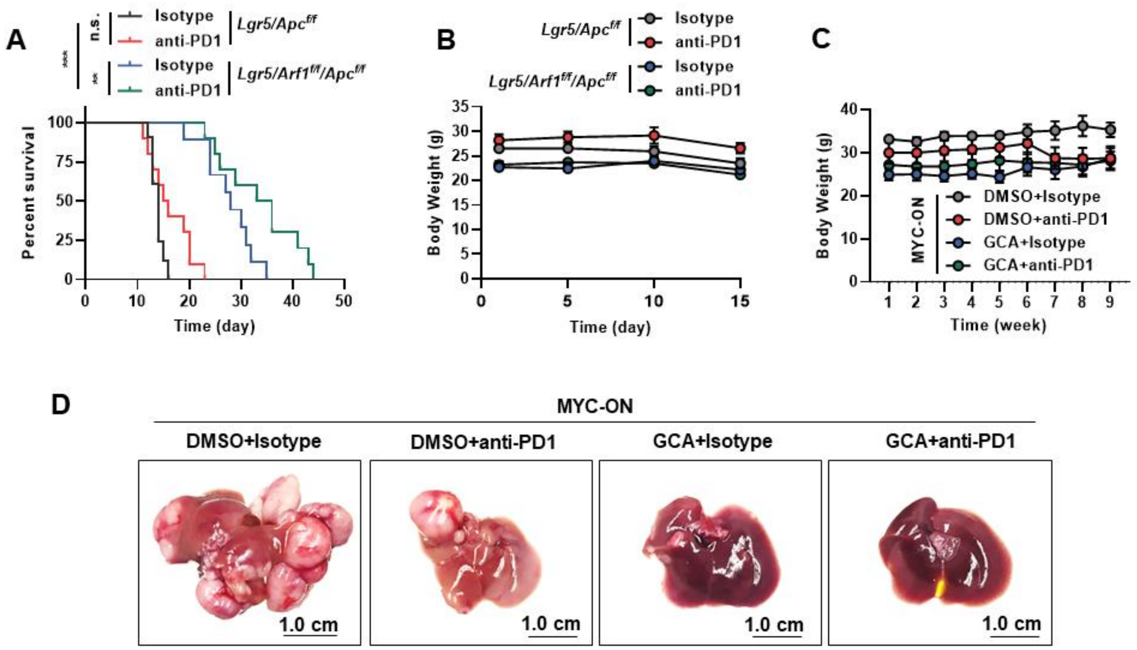

Supplementary Figure 15. Arf1 ablation and PD1 blockage have synergistic anti-tumor effects. (A-B) Percent survival and body weight curves of intestinal tumor mice of the indicated genotypes after treatment with isotype or anti-PD1 antibody (n=10 mice each group, pooled two independent experiments). (C) Body weight curves of MYC-ON mice treated with the indicated reagents (n=10 mice each group, pooled two independent experiments). (D) Representative liver images of MYC-ON mice treated with the indicated reagents. Scale bars: 1.0 cm. Data are shown as the mean +/- SEM. **$p<0.01$, ***$p<0.001$ by Student's t test. Scale bars are as indicated.

Supplementary Figure 16

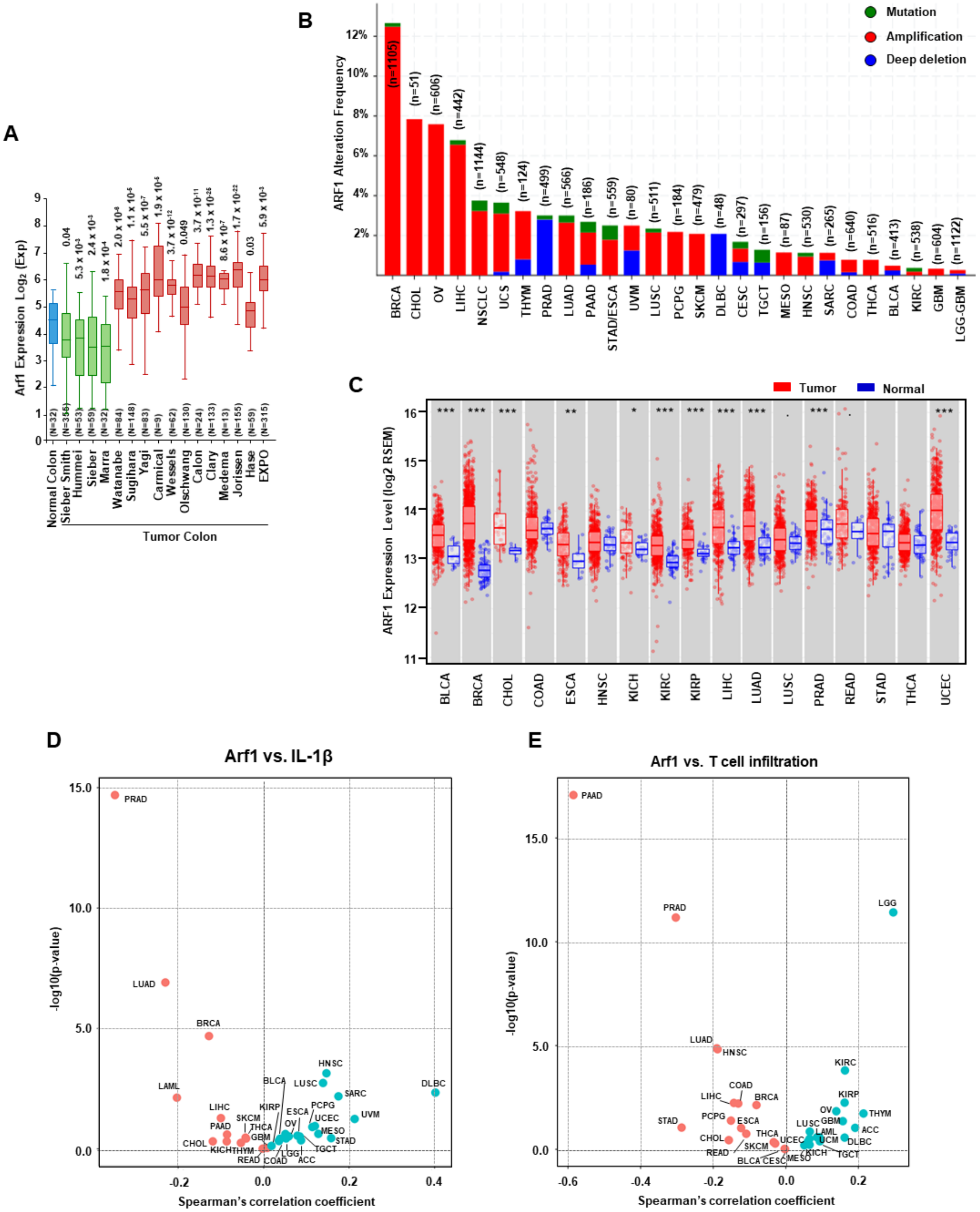

Supplementary Figure 16. The frequencies of mutation, amplification, and deletion of Arf1 in human cancers. (A) Analysis of Arf1 expression in normal and tumor colons from patients with the

indicated names the R2 Genomics Analysis and Visualization Platform. (B) Arf1 alterations in a panel of cancer types analyzed using cBioPortal. (C) Analysis of Arf1 expression in normal and tumor tissues from patients with the indicated types of cancers in the TCGA dataset. (D) Correlation analysis for the Arf1 expression level versus the IL-1β signature in tumors from the indicated types of cancer patients in the TCGA dataset. (E) Correlation analysis for the Arf1 expression level versus the CD8α+ T cell infiltration in tumors from the indicated types of cancer patients in the TCGA dataset.

Supplementary Figure 17

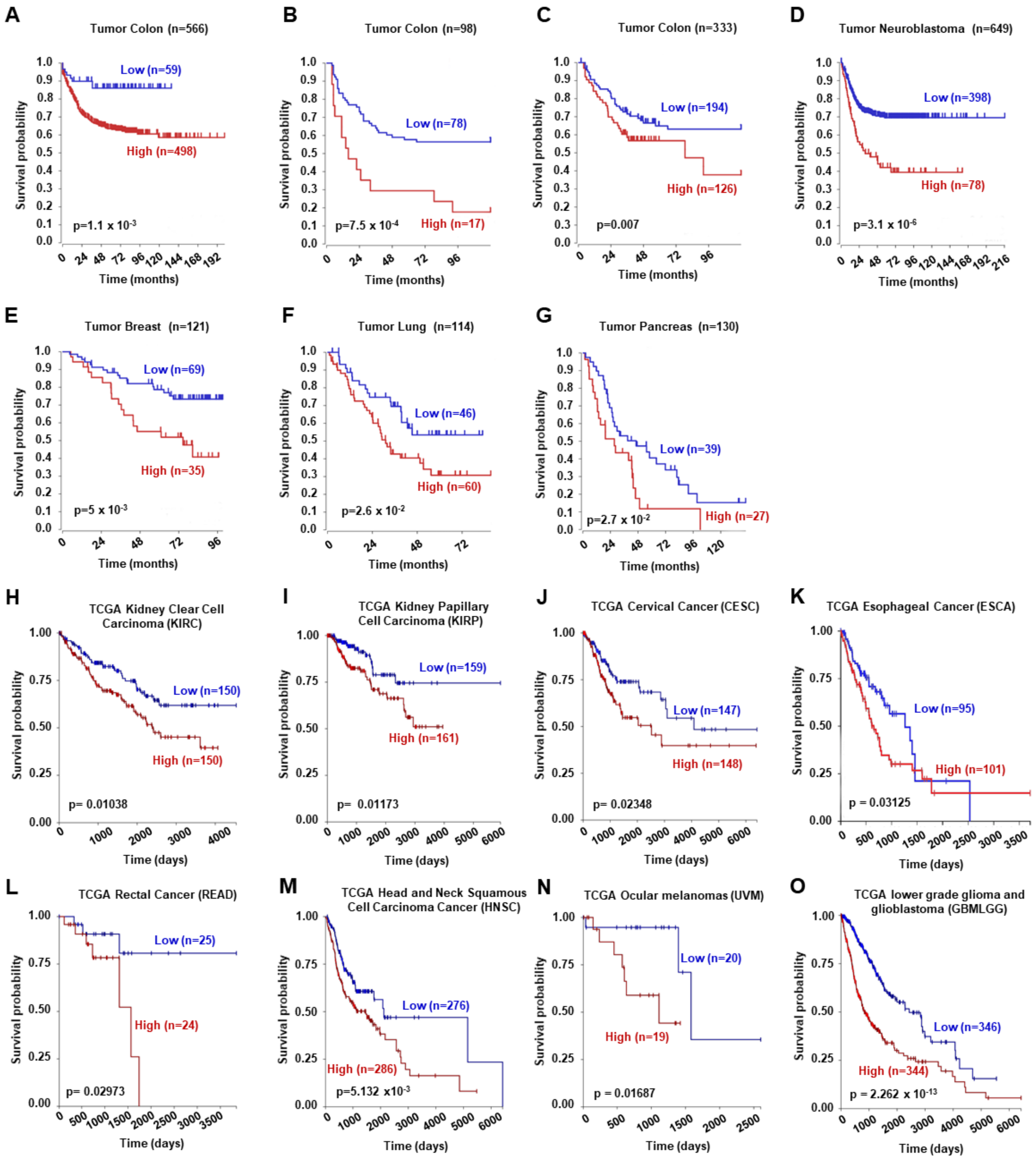

Supplementary Figure 17. Survival curves of Arf1-low (blue) and Arf1-high (red) patient groups. The figures were generated with the indicated types of tumors using the R2 genomics analysis and visualization platform and TCGA dataset.

Supplementary Figure 18

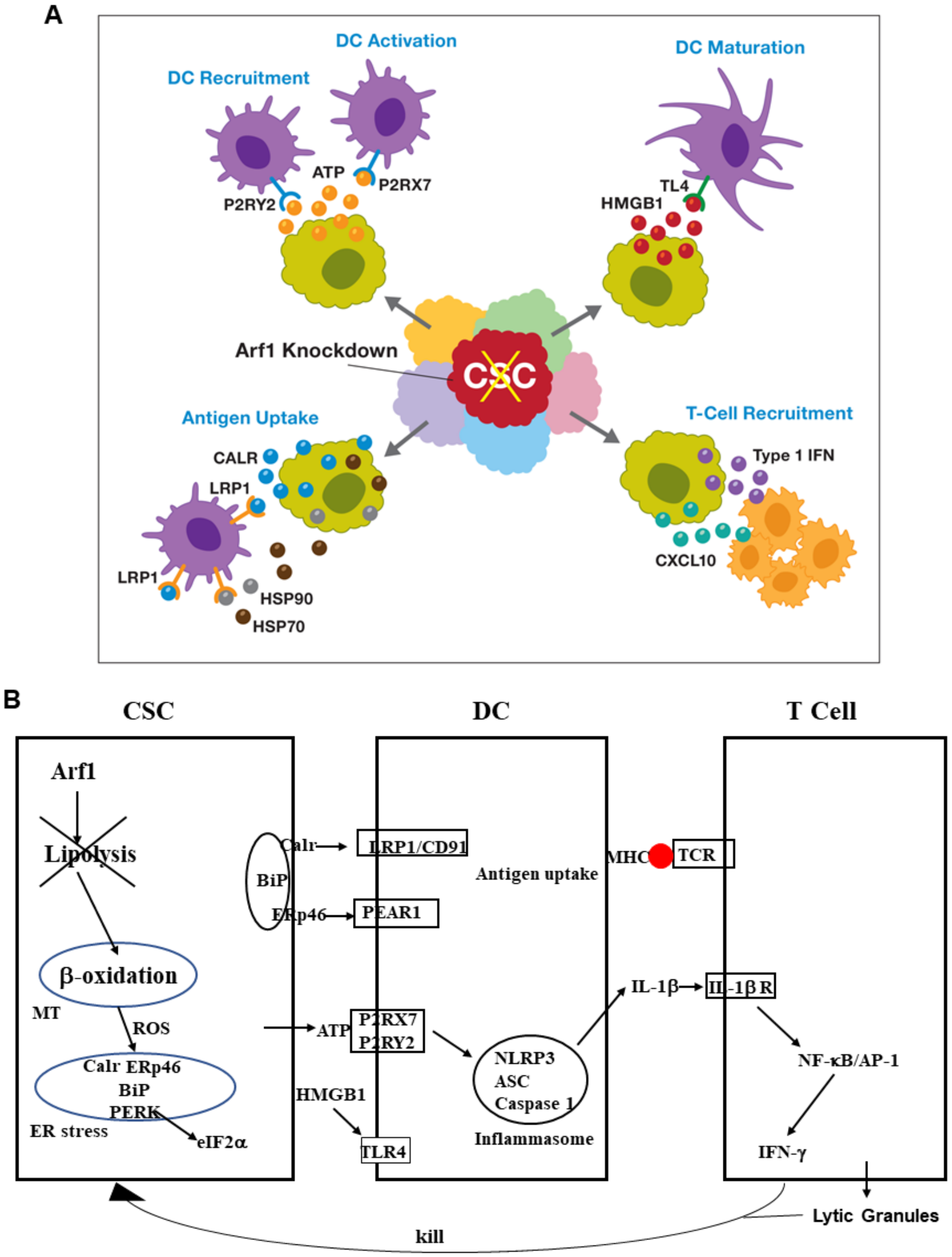

Supplementary Figure 18. Proposed models. (A) Proposed model depicting how Arf1 ablation induces DAMP-mediated, anti-tumor immune responses. (B) Proposed Model depicting the molecular pathway by which Arf1 ablation induces DAMP-mediated anti-tumor immune responses.

Supplementary Figure 19

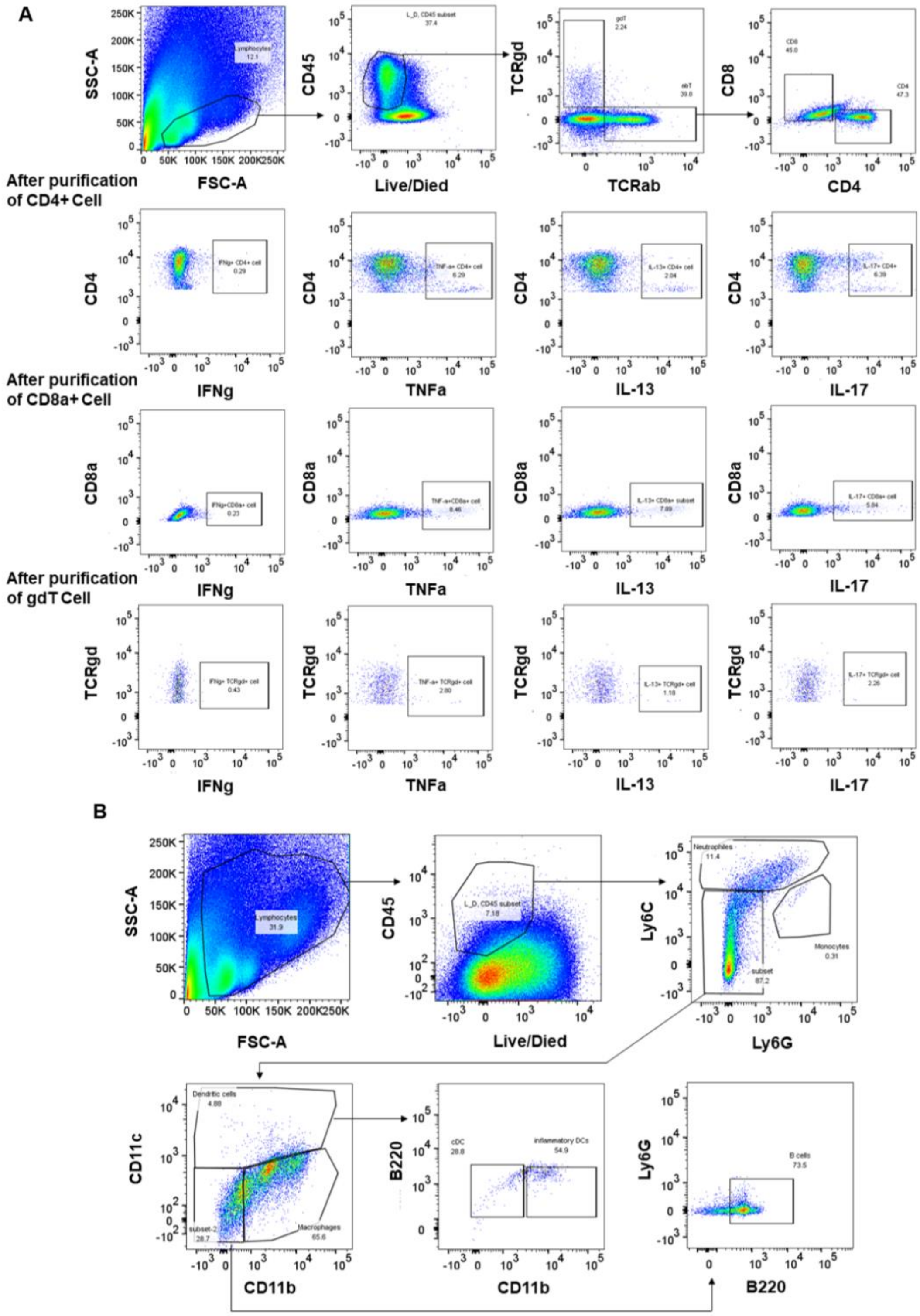

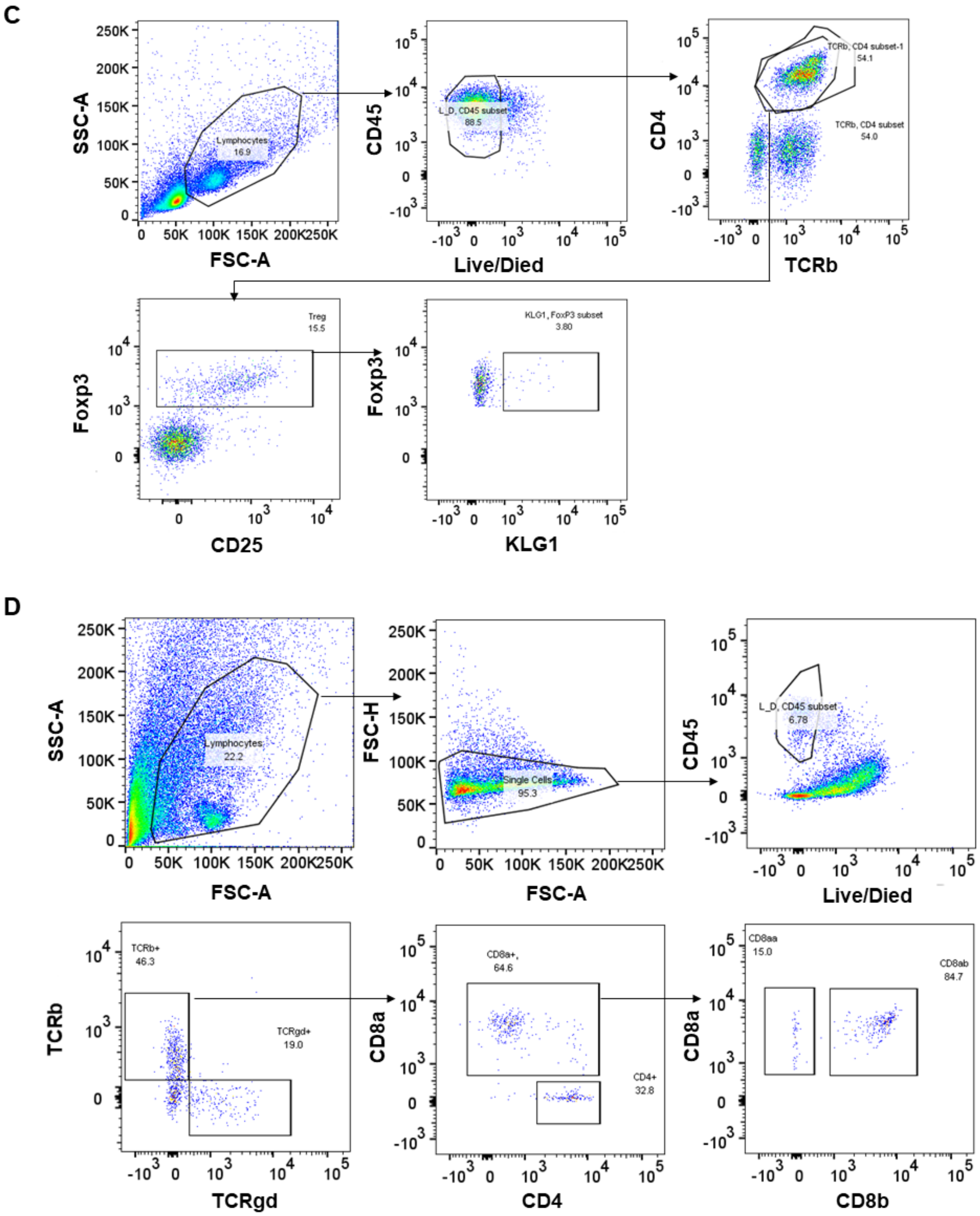

Supplementary Figure 19. Gating strategies used for sorting the intestine immune cell. (A) Gating strategy for sort the CD4+ (CD45+CD4+TCRab+) cells, CD8a+ (CD45+CD8a+TCRab+) cells, gdT+ (CD45+TCRgd+) cells, CD4+TNFa+ (CD45+CD4+TCRab+TNFa+) cells, CD4+IFNg+ (CD45+CD4+TCRab+IFNg+) cells, CD4+IL-13+ (CD45+CD4+TCRab+IL-13+) cells, CD4+IL-17+ (CD45+CD4+TCRab+IL-17+) cells, CD8a+TNFa+ (CD45+CD8a+TCRab+TNFa+) cells, CD8a+IFNg+ (CD45+CD8a+TCRab+IFNg+) cells, CD8a+IL-13+ (CD45+CD8a+TCRab+IL-13+) cells, CD8a+IL-17+

(CD45+CD8a+TCRab+IL-17+) cells, gdT+TNFa+ (CD45+TCRgd+TNFa+) cells, gdT+IFNg+ (CD45+TCRgd+IFNg+) cells, gdT+IL-13+ (CD45+TCRgd+IL-13+) cells and gdT+IL-17+ (CD45+TCRgd+IL-17+) cells from control and Lgr5/Arf1/Apc mice for presented on the figure 2 and supplementary figure 4. (B) gating strategy for sort neutrophils (CD45+Ly6c+), monocytes (CD45+Ly6g+), macrophage (CD45+CD11b+), B cell (CD45+B220+), dendritic cell (CD45+CD11c+) and inflammatory dendritic cells (CD45+CD11c+CD11b+) from mouse intestine for presented on the figure 2 and supplementary figure 4. (C) gating strategy for sort Treg+ cells (CD45+CD4+CD25+Foxp3+) in the mouse intestine for presented on the figure 2 and supplementary figure 4. (D) Gating strategy of mouse intestine IEL gdT cells (CD45+TCRgd+), CD8aa+ cells (CD45+CD8a+TCRab+) and CD8ab+ cells (CD45+CD8a+CD8b+TCRab+) for presented on the figure 2 and supplementary figure 4.

Supplementary Figure 20

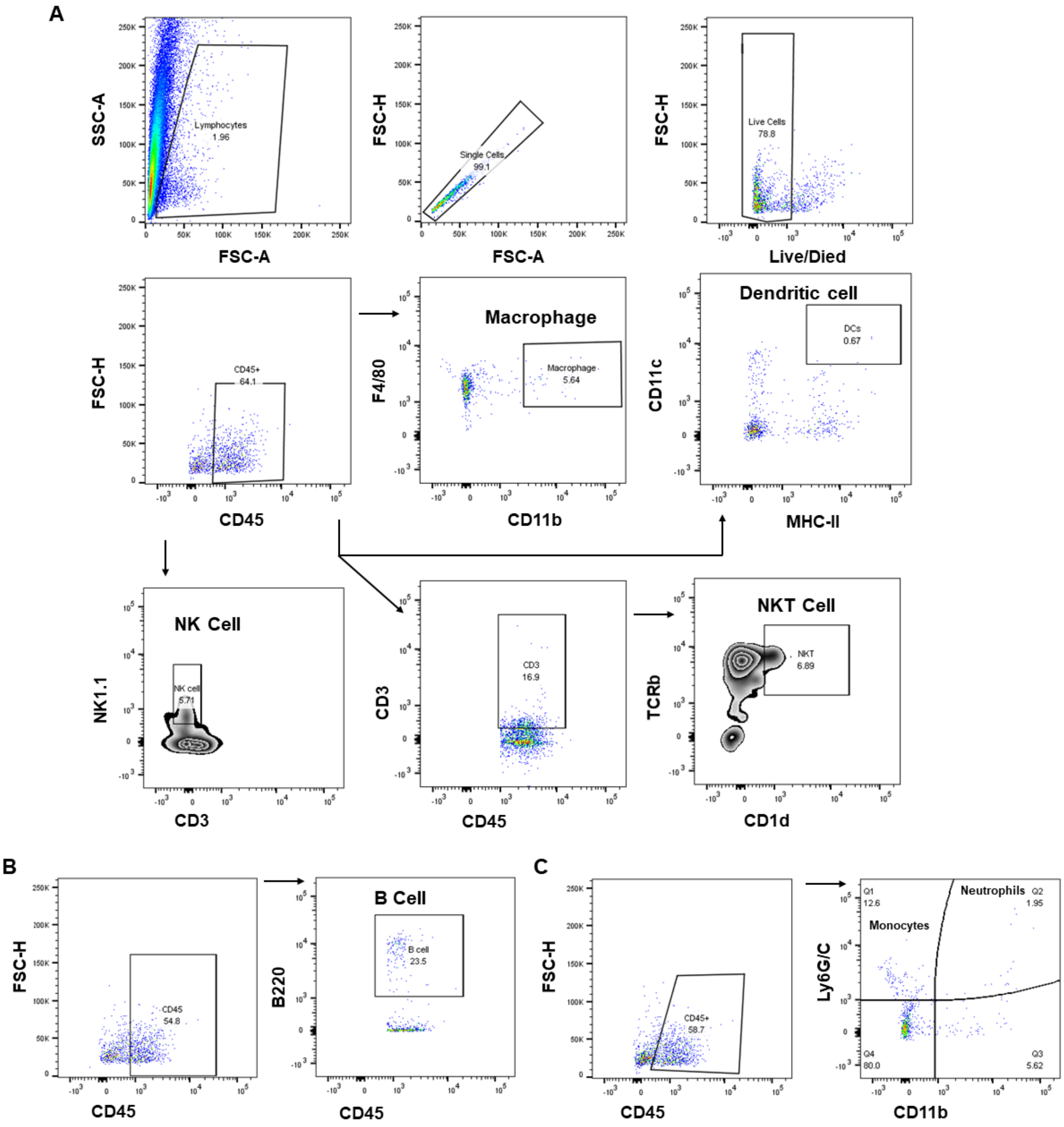

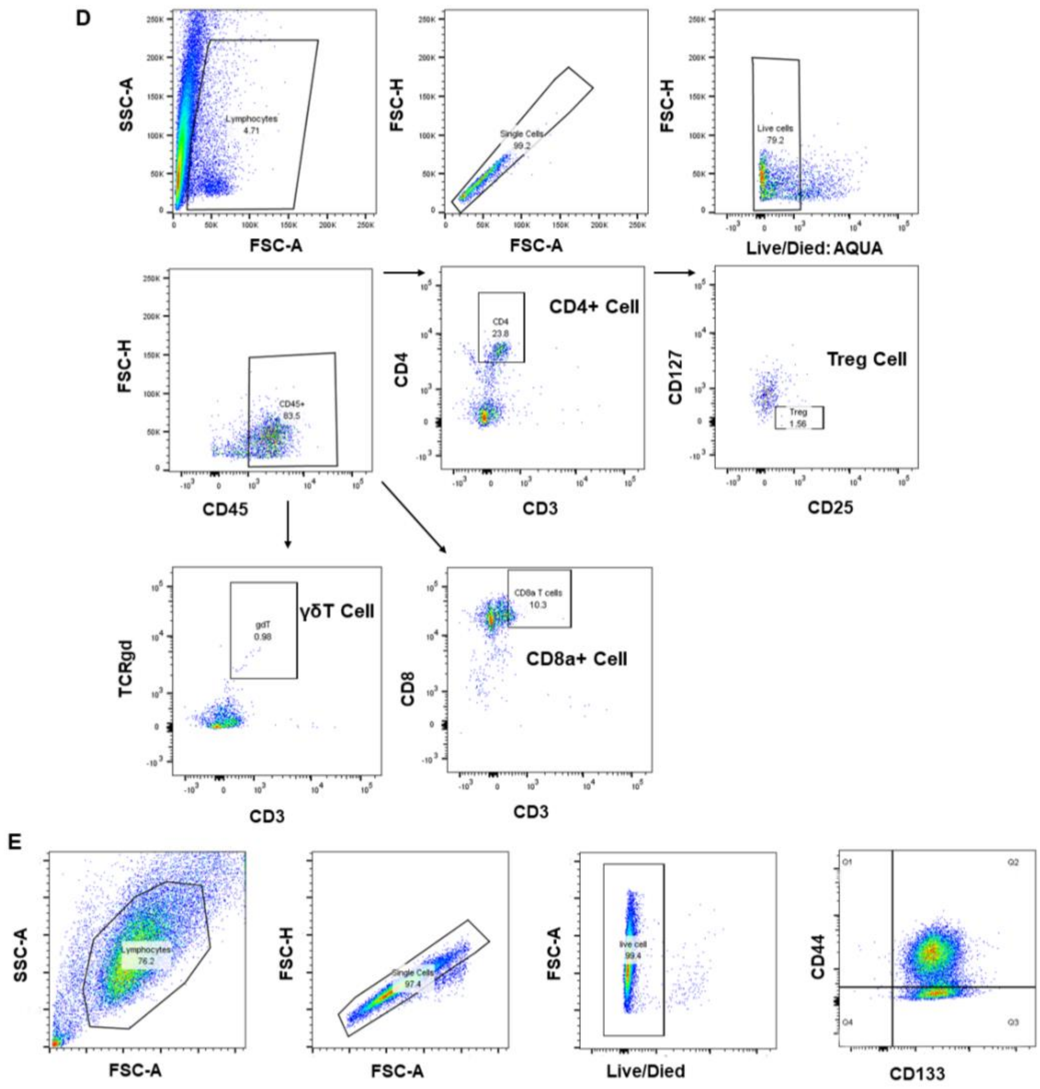

Supplementary Figure 20. Gating strategies used for sorting the liver immune cell. (A) Gating strategy for sort the Macrophages (CD45+CD11b+F4/80+), dendritic cells (CD45+CD11c+MHC-II+), NK cells (CD45+CD3-NK1.1+) and NKT cells (CD45+CD3+TCRb+Cd1d+). (B) Gating strategy for sort B cell (CD45+B220+) from liver. (C) Gating strategy for sort monocytes (CD45+CD11b+Ly6g/c-) and neutrophils (CD45+CD11b+Ly6g/c+) from liver. (D) Gating strategy for sort CD4+ T cell (CD45+CD3+CD4+), Treg (CD45+CD3+CD4+CD25+CD127-), gdT cell (CD45+CD3+TCRgd+) and CD8+ T cells (CD45+CD3+CD8a+) from liver for presented on the supplementary figure 5D. (E) Gating strategy of in vitro isolation of cancer stem cells (CD133+CD44+) from 4T1, B16-F10 and CT26 cells for presented on the supplementary figure 9E.

Supplementary Figure 21

A

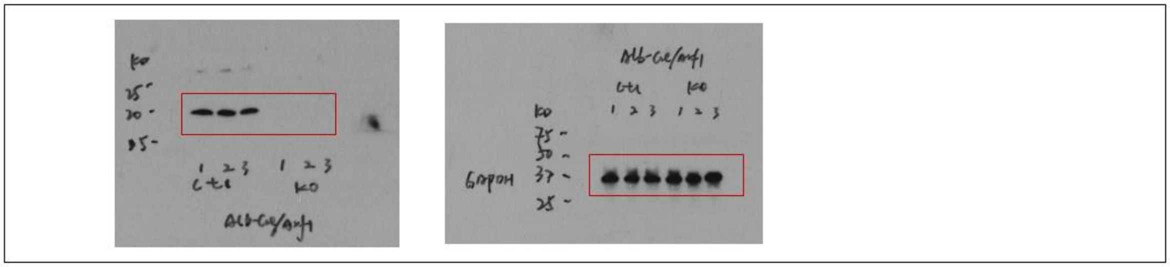

B

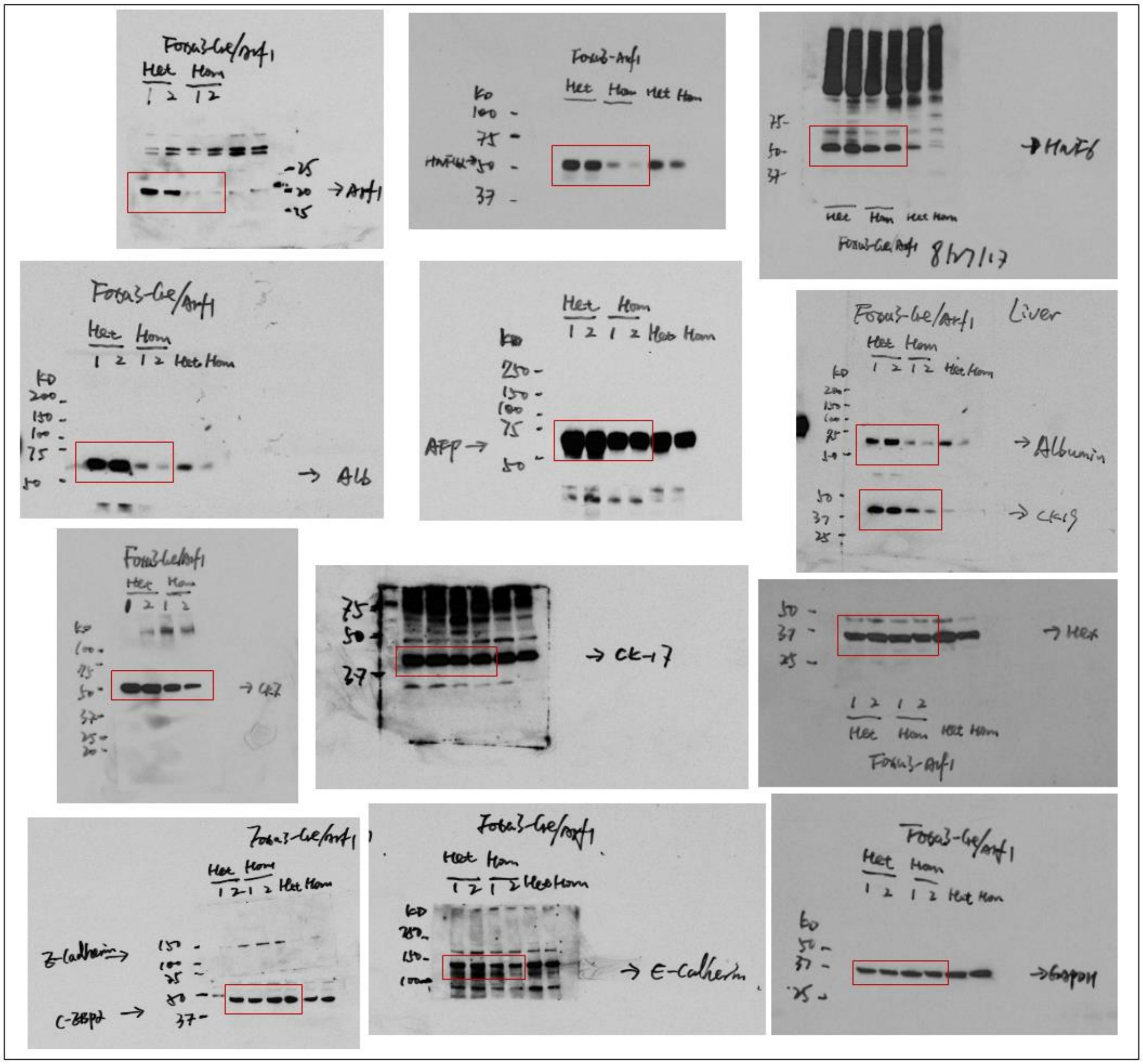

C

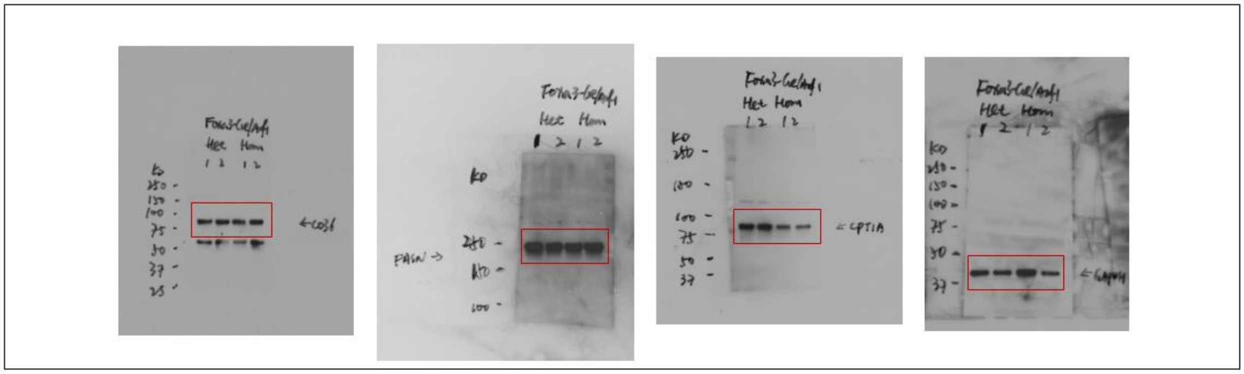
Het Hom
FASN
CPT1A
GAPDH

D

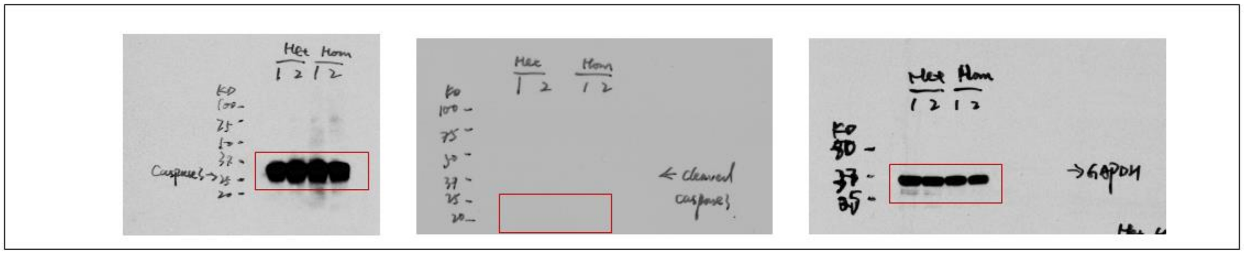
Het Hom
Caspase3
cleaved caspase3
GAPDH

E

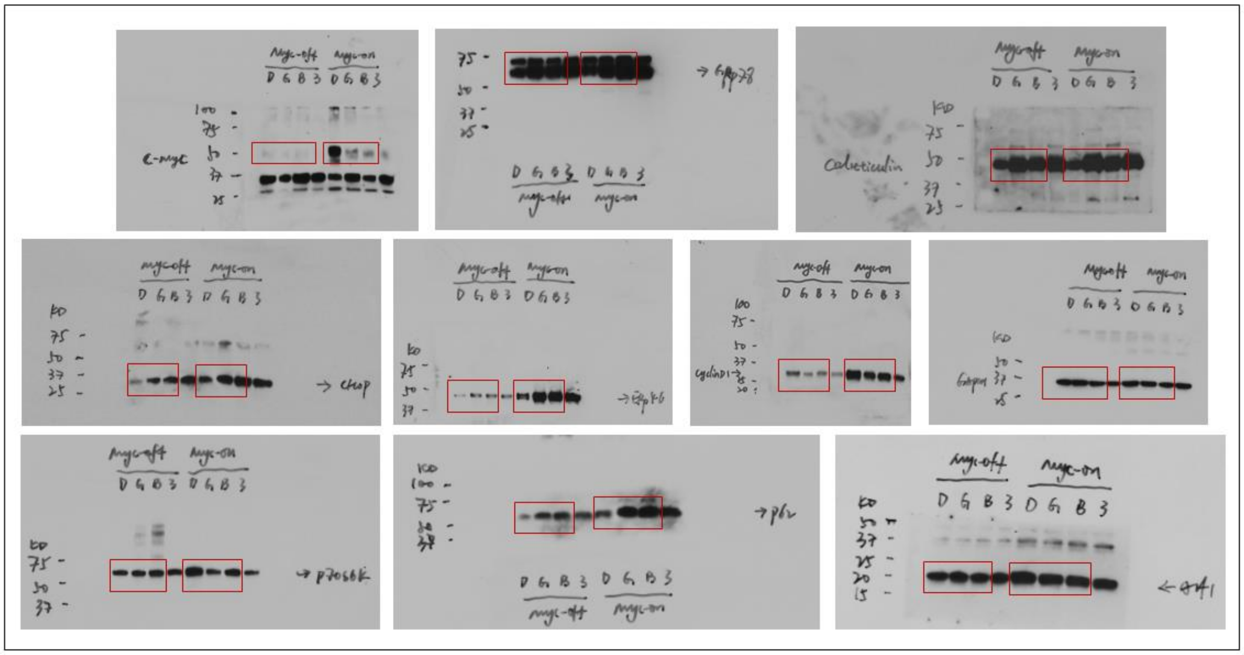
Myc-off Myc-on
D G B 3 D G B 3
c-myc
Grp78
Calreticulin
chop
Cyclin D1
Gapdh
p70S6K
p62

F

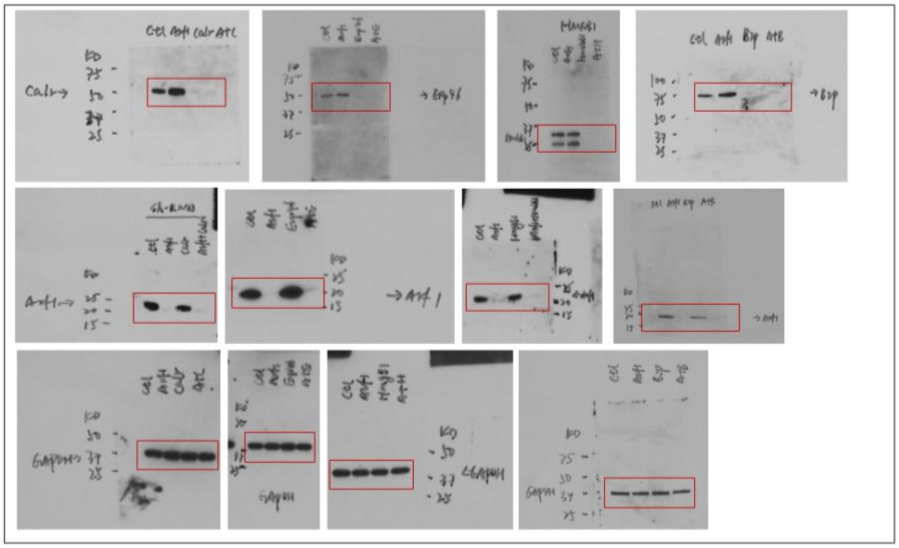

G

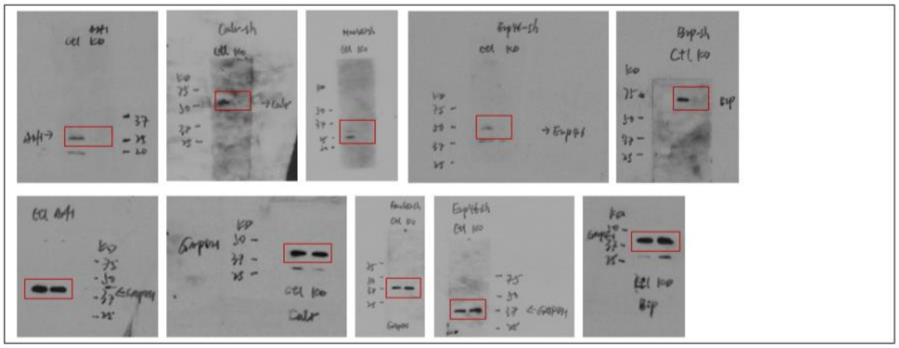

H

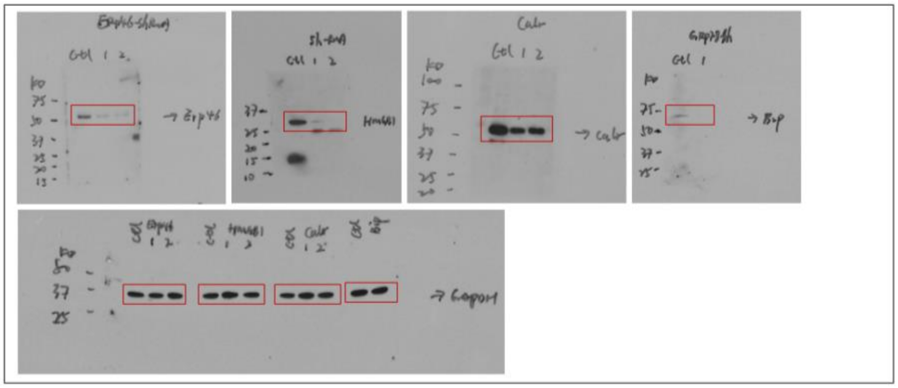

Supplementary Fig. 21 Uncropped western blotting images for the results shown in supplementary figures. (A-C) Represent the full-length western blotting images of Supplemental Fig. 2c (A), Supplemental Fig. 2I (B), Supplemental Fig. 2N (C). (D) The uncut western blot images for supplemental Fig. 7a. (E) The full-length western blot images for supplemental Fig.8I. (F-H) Represent the full-length western blotting images of Supplemental Fig.11a (F), Supplemental Fig.12a (G) and Supplemental Fig.13a (H).

Supplementary Table

Supplementary Table 1: Primers used in this paper

Primer	Sequence
Ccl2-F	5’-TCATGCTTCTGGGCCTGCTG-3’
Ccl2-R	5’-CTTTGGGACACCTGCTGCTG-3’
Ccl3-F	5’-CACTGCCCTTGCTGTTCTTC-3’
Ccl3-R	5’-CACCTGGCTGGGAGCAAAGG-3’
Ccl5-F	5’-CACCATCATCCTCACTGCAG-3’
Ccl5-R	5’-CTTCGAGTGACAAACACGAC-3’
Ccl17-F	5’-CTTCACCTCAGCTTTTGGTA-3’
Ccl17-R	5’-TACCAGCTCACCAACTTCCTG-3’
Ccl20-F	5’-GTCTGCTCTTCCTTGCTTTG-3’
Ccl20-R	5’-TGATAGCATTAATGTCACAAG-3’
Ccl28-F	5’-GCAGCAAGCAGGGCTCACA-3’
Ccl28-R	5’-GGATGACAGCAGCCAGGTCG-3’
Cxcl10-F	5’-TGAACCCAAGTGCTGCCGTC-3’
Cxcl10-R	5’-CATCGTGGCAATGATCTCAA-3’
Cxcl11-F	5’-GTCACAGCCATAGCCCTGGC-3’
Cxcl11-R	5’-CCTTCATAGTAACAATCACT-3’
IL6-F	5’-GCAAGAGACTTCCATCCAG-3’
IL6-R	5’-CAGAATTGCCATTGCACAAC-3’
TNFα-F	5’-CTACACAGAAGTTCCCAAAT-3’
TNFα-R	5’-CCCTTTCCTCCCAAACCAA-3’
Ccl4-F	5’-GAAGCTCTGCGTGTCTGCCCT-3’
Ccl4-R	5’-CCACAGCTGGCTTGGAGCAA-3’
Ccl22-F	5’-GGCTACCCTGCGTGTCCCACT-3’
Ccl22-R	5’-GGTCCAGAAGAACTCCTTCA-3’
Cxcl9-F	5’-GCATCATCTTCCTGGAGCA-3’
Cxcl9-R	5’-CTAGGCAGGTTTGATCTCCG-3’
Gapdh-F	5’-GTCCCAGCTTAGGTTCATCA-3’
Gapdh-R	5’-GAGGTCAATGAAGGGGTCGT-3’
IFNγ-F	5’-GGTGACATGAAAATCCTGCAGA-3’
IFNγ-R	5’-GCAATACTCATGAATGCATCC-3’
IL-1β-F	5’-GCTGCTTCCAAACCTTTGACC-3’
IL-1β-R	5’-GGTGCTCATGTCCTCATCCTGG-3’
Perforin-F	5’-CACTCGGTCAGAATGCAAGCA-3’
Perforin-R	5’-GTGGCGTCTCTCATTAGGGAGT-3’
GzmA-F	5’-GCAACAGTTAACAGAAATGTGGC-3’
GzmA-R	5’-CATCATTGCAGATTTTTCTGTCT-3’
GzmB-F	5’-GAAGAGTAAGGCCAAGAGGACTA-3’
GzmB-R	5’-CTCACACTCCCGATCCTTCTGT-3’
PD-L1-F	5’-GCATTATATTCACAGCCTGCTG-3’
PD-L1-R	5’-TGCCACAAACTGAATCACTTGC-3’
ERp46-Exon3-F	5’-<br>CCGGTTGCTGTTGACAGTGAGCGACTGCAGACACTGAACGAGGAGTAGTGAAGCCACAGATGTACT

	CCTCGTTCAGTGTCTGCAGCTGCCTACTGCCTCGGAG-3’
ERp46-Exon3-R	5’-AATTCTCCGAGGCAGTAGGCAGCTGCAGACACTGAACGAGGAGTACATCTGTGGCTTCACTACTCCTCGTTCAGTGTCTGCAGTCGCTCACTGTCAACAGCAA-3’
ERp46-Exon2-F	5’-CCGGTTGCTGTTGACAGTGAGCGCACTTGGAATGACCTGGGAGACTAGTGAAGCCACAGATGTAGTCTCCCAGGTCATTCCAAGTTTGCCTACTGCCTCGGAG-3’;
ERp46-Exon2-R	5’-AATTCTCCGAGGCAGTAGGCAAACTTGGAATGACCTGGGAGACTACATCTGTGGCTTCACTAGTCTCCCAGGTCATTCCAAGTGCGCTCACTGTCAACAGCAA-3’
Hmgb1-Exon2-F	5’-CCGGTTGCTGTTGACAGTGAGCGCTGTCCTCATATGCATTCTTTGTAGTGAAGCCACAGATGTACAAAGAATGCATATGAGGACATTGCCTACTGCCTCGGAG-3’
Hmgb1-Exon2-R	5’-AATTCTCCGAGGCAGTAGGCAATGTCCTCATATGCATTCTTTGTACATCTGTGGCTTCACTACAAAGAATGCATATGAGGACAGCGCTCACTGTCAACAGCAA-3’
Hmgb1-Exon3-F	5’-CCGGTTGCTGTTGACAGTGAGCGAGCTGACAAGGCTCGTTATGAATAGTGAAGCCACAGATGTATTCATAACGAGCCTTGTCAGCCTGCCTACTGCCTCGGAG-3’
Hmgb1-Exon3-R	5’-AATTCTCCGAGGCAGTAGGCAGGCTGACAAGGCTCGTTATGAATACATCTGTGGCTTCACTATTCATAACGAGCCTTGTCAGCTCGCTCACTGTCAACAGCAA-3’
GRp78-Exon2-F	5’-CCGGTTGCTGTTGACAGTGAGCGACGCGTGGAGATCATAGCCAACTAGTGAAGCCACAGATGTAGTTGGCTATGATCTCCACGCGGTGCCTACTGCCTCGGAG-3’
GRp78-Exon2-R	5’-AATTCTCCGAGGCAGTAGGCACCGCGTGGAGATCATAGCCAACTACATCTGTGGCTTCACTAGTTGGCTATGATCTCCACGCGTCGCTCACTGTCAACAGCAA-3’
Cpt1a-F	5’-AGGCTCACCAAGCTGTGGCC-3’
Cpt1a-R	5’-TAAGCCAGCTGGAGGGACTC-3’
FASN-F	5’-AGGTGGTGATAGCCGGTATG-3’
FASN-R	5’-GGCGTCGAACTTGGAGAGAT-3’
CD36-F	5’-AAGTTGTCCTTGAAGAAG-3’
CD36-R	5’-AGATAACGAACTCTGTATGTG-3’
HIF1a-F	5’-AGGATGAGTTCTGAACG-3’
HIF1a-R	5’-CGGCATCCAGAAGTTTTCTC-3’
STAT1-F	5’-ATGGTAGCACGCAACCAGGT-3’
STAT1-R	5’-GTGCGGAAGTGAGTGTCGCC-3’
MYC-F	5’-ATGCCCCTCAACGTGAACTT-3’
MYC-F	5’-GAAGCAGCTCGAATTTCTTC-3’
AP1-F	5’-ATGACTGCAAAGATGGAAACG-3’
AP1-R	5’-CTTGGCGCGGAGGTGCGGCTTC-3’
NFkb1-F	5’-ATGGCAGACGATGATCCCTA-3’
NFkb1-R	5’-TGATGGGCCTTCACACACATA-3’
Arf1-siRNA-1-F	5’-GAGACUGUUGAAUACAAGAAUAU-3’
Arf1-siRNA-1-R	5’-AUUCUUGUAUUCAACAGUCUCCA-3’
Arf1-siRNA-2-F	5’-UCUCUUGGUGUUUGCCAACAAG-3’

Arf1-siRNA-2-R	5’-UGUUGGCAAACACCAAGAGAAC-3’
