# Supplementary material for: Arf1-mediated lipid metabolism sustains cancer cells and its ablation induces anti-tumor immune responses in mice
Source: Nat Commun. 2020 Jan 10;11:220. doi: 10.1038/s41467-019-14046-9 (PMC6954189; doi:10.1038/s41467-019-14046-9)
Supplement: Supplementary file 3 — Reporting Summary [file 41467_2019_14046_MOESM3_ESM.pdf]

nature research

Corresponding author(s): Steven X. Hou

Last updated by author(s): Nov 9, 2019

Reporting Summary

Nature Research wishes to improve the reproducibility of the work that we publish. This form provides structure for consistency and transparency in reporting. For further information on Nature Research policies, see Authors & Referees and the Editorial Policy Checklist.

Statistics

For all statistical analyses, confirm that the following items are present in the figure legend, table legend, main text, or Methods section.

n/a	Confirmed	
☐	☒	The exact sample size (n) for each experimental group/condition, given as a discrete number and unit of measurement
☐	☒	A statement on whether measurements were taken from distinct samples or whether the same sample was measured repeatedly
☐	☒	The statistical test(s) used AND whether they are one- or two-sided<br>*Only common tests should be described solely by name; describe more complex techniques in the Methods section.*
☒	☐	A description of all covariates tested
☐	☒	A description of any assumptions or corrections, such as tests of normality and adjustment for multiple comparisons
☐	☒	A full description of the statistical parameters including central tendency (e.g. means) or other basic estimates (e.g. regression coefficient) AND variation (e.g. standard deviation) or associated estimates of uncertainty (e.g. confidence intervals)
☐	☒	For null hypothesis testing, the test statistic (e.g. F, t, r) with confidence intervals, effect sizes, degrees of freedom and P value noted<br>*Give P values as exact values whenever suitable.*
☒	☐	For Bayesian analysis, information on the choice of priors and Markov chain Monte Carlo settings
☒	☐	For hierarchical and complex designs, identification of the appropriate level for tests and full reporting of outcomes
☐	☒	Estimates of effect sizes (e.g. Cohen's d, Pearson's r), indicating how they were calculated

Our web collection on statistics for biologists contains articles on many of the points above.

Software and code

Policy information about availability of computer code

Data collection	Immunofluorescence were taken by Zeiss 710 and 780 confocal microscopy, ZEN software were used to analysis the images. FolwJo_V10 were used to analysis the FACS plots. qRT-PCR were performed by Bio-Rad C100 Touch Thermal Cycler and analysis by Bio-Rad CFX XI software, each each group use three to five replicates. For human cancer patients dataset, the data from The Cancer Genome Atlas data set from NCI and R2: Genomics Analysis and Visualization Platform.
Data analysis	Human cancer patients results were analyzed by R2: Genomics Analysis and Visualization Platform, for colon tumor Colon CIT (combat)-Marisa-566-rma-u133p2, Tumor Colon (Core-Transcript)-Sveen-333-rma_sketch-huex10t and Tumor Colon MSI-status (Core-Transcript)-Sveen-95-rma_sketch-huex10t datasets were use to analysis with Kaplan scan a single gene:Arf1 and the cutoff_modus is Scan. For Neuroblastoma, the Tumor Neuroblastoma-Kocak-649-custom-ag44kcwolf were use to analysis the Arf1 expression with the patient survival. The Breast tumor of Arf1 expression level in patients was analyzed by Mix Tumor Breast-Clynes-121-MAS5.0-u133p2 dataset. The Lung tumor of Arf1 expression level in patients was analyzed by Tumor Lung-Bild-114-MAS5.0-u133p2 dataset. The Pancreas tumor of Arf1 expression level in patients was analyzed by Mix Tumor Pancrea (Transcript)-Hussain-130-rma_sketch-hugene10t dataset.<br>For using TCGA to analysis the Arf1 expression level, the TCGA Kidney Clear Cell Carcinoma (KIRC), TCGA Kidney Papillary Cell Carcinoma (KIRP), TCGA Cervical Cancer (CESC), TCGA Esophageal Cancer (ESCA), TCGA Rectal Cancer (READ), TCGA Head and Neck Squamous Cell Carcinoma Cancer (HNSC), TCGA Ocular melanomas (UVM), TCGA lower grade glioma and glioblastoma (GBMLGG) were used to analysis the Arf1 expression level for compare the survival time of patients.<br>Arf1 levels between a human patient's tumor and the adjacent normal tissues across the TCGA database was analyzed by the TIMER.<br>The ARF1 expression levels vs IL-1β and IFNγ were analyzed in The Cancer Genome Atlas data set (NCI, NIH) by the Cbioportal website (http://www.cbioportal.org/) using Spearman's correlations.

For manuscripts utilizing custom algorithms or software that are central to the research but not yet described in published literature, software must be made available to editors/reviewers. We strongly encourage code deposition in a community repository (e.g. GitHub). See the Nature Research guidelines for submitting code & software for further information.

Data

Policy information about availability of data

All manuscripts must include a data availability statement. This statement should provide the following information, where applicable:

- Accession codes, unique identifiers, or web links for publicly available datasets
- A list of figures that have associated raw data
- A description of any restrictions on data availability

All the data supporting the findings of this study are available within the article and its supplementary information files and from the corresponding author upon reasonable request. A reporting summary for this article is available as a Supplementary Information file. The source data underlying Figures and Supplementary Figures are provided as a Source Data file.

Field-specific reporting

Please select the one below that is the best fit for your research. If you are not sure, read the appropriate sections before making your selection.

☒ Life sciences ☐ Behavioural & social sciences ☐ Ecological, evolutionary & environmental sciences

For a reference copy of the document with all sections, see nature.com/documents/nr-reporting-summary-flat.pdf

Life sciences study design

All studies must disclose on these points even when the disclosure is negative.

Sample size	Most of the samples were list in the experiment method,<br>For mice intestine tumor number, each group mice is 15. The intestine tumor mice survival, each group is 15 mice.<br>For the intestine tumor immune cells analysis, each group use 3 to 5 mice.<br>For electron microscopy, each group use 2 or 3 mice. For qRT-PCR, each group were use 5 mice.<br>For liver tumor model, each group use 5 to 8 mice to calculate the tumor number, and the survival cure use 8 to 12 mice of each group.<br>For antibody treatment of MYC-On mice, each group use 9 to 10 mice. The Lgr5/Arf1/Apc intestine tumor mice, each group use 5 mice.<br>The inhibitors treated mice, each group use 5 mice.<br>The xenograft and transplant experiment, each group use 9 to 10 mice.<br>For immunohistology experiments, at least 3 mice tissue were processed for section and stained with antibodies. For each staining, at least 10 images were taken randomly, one image were shown in the figure.<br>For Western blot analysis, all of the experiments were repeated 3 times from protein preparation and 1 representative blot was shown in the figure.
Data exclusions	No data were excluded from the analyses.
Replication	All attempts at replication were successful.
Randomization	All the mice were separate for different groups are randomization. most of mice were tried use their littermates as control.
Blinding	Tumor size monitor and IHC, IF Images were taken by person who was blinded to group allocation.

Reporting for specific materials, systems and methods

We require information from authors about some types of materials, experimental systems and methods used in many studies. Here, indicate whether each material, system or method listed is relevant to your study. If you are not sure if a list item applies to your research, read the appropriate section before selecting a response.

Materials & experimental systems

n/a	Involved in the study
☐	☒ Antibodies
☐	☒ Eukaryotic cell lines
☒	☐ Palaeontology
☐	☒ Animals and other organisms
☒	☐ Human research participants
☒	☐ Clinical data

Methods

n/a	Involved in the study
☒	☐ ChIP-seq
☐	☒ Flow cytometry
☒	☐ MRI-based neuroimaging

Antibodies

Antibodies used	For IHC, IF and WB:<br>Mouse anti-β-Catenin (BDI080), Abcam, Cat#Ab19448;

Chicken anti-GFP, Abcam, Cat#Ab13970;
Rabbit anti-CD3ε (D4V8L), Cell Signaling, Cat#99940;
Rabbit anti-CD4 [EPR19514], Abcam, Cat# ab183685;
Rat anti-CD8a Antibody (4SM15), eBioscience, Cat#14-0808-82;
Rabbit anti-pZap-70 (65E4), Cell Signaling, Cat#2717;
Mouse anti-mouse I-AK (AαK) (MHC-II) (11-5.2), Biolegend, Cat#110002;
Mouse anti-mouse H-2Kb/H-2Db, Biolegend, Cat#114602;
Rabbit anti-Calreticulin Antibody, ThermoFisher, Cat#PA3-900;
Mouse anti-ERp46 Antibody (C-11), Santa Cruz, Cat#sc-271667;
Rabbit anti-HMGB1 Polyclonal Antibody, ThermoFisher, Cat#PA1-16926;
Mouse anti-human LAMP-1 Antibody (H4A3), Biolegend, Cat#328601;
Rabbit anti-eIF2A [pSer51] Antibody, Novus Biologicals, Cat#NB100-81896;
Rabbit anti-Phospho-eIF2α (Ser51), Cell Signaling, Cat#9721;
Rabbit anti-Cleaved Caspase-3 (Asp175) (5A1E), Cell Signaling, Cat#9664S;
Mouse anti-Caspase-1 (p20), AdipoGen Life Science, Cat#AG-20B-0042-C100;
Rabbit anti-PEAR1 Polyclonal Antibody, ThermoFisher, Cat#PA5-21057;
Armenian Hamster anti-CD11c Antibody (AP-MAB0806), Novus Biologicals, Cat#NB110-97871;
Mouse anti-LRP1 Monoclonal Antibody, ThermoFisher, Cat#37-3800;
Mouse anti-KDEL (10C3), Novus Biologicals, Cat#NBP1-97469;
Mouse anti-HNF-4-alpha antibody [K9218], Abcam, Cat#ab41898;
Rabbit anti-alpha 1 Fetoprotein antibody, Abcam, Cat#ab46799;
Rabbit anti-Albumin antibody [EPR20195], Abcam, Cat#ab207327;
Mouse anti-HNF-6 Antibody (G-10), Santa Cruz, Cat#sc-376167;
Mouse anti-Cytokeratin 19 Antibody (A-3), Santa Cruz, Cat#sc-376126;
Mouse anti-Hex antibody, Abcam, Cat#Ab117864;
Rabbit anti-ARF1 Polyclonal Antibody, ThermoFisher, Cat#PA1-127;
Mouse anti-GAPDH Antibody (GA1R), ThermoFisher, Cat#MA5-15738;
Mouse Anti-E Cadherin antibody [M168], Abcam, Cat#Ab76055;
Rabbit anti-CEBP Alpha/CEBPA antibody, Abcam, Cat#Ab40764;
Rabbit anti-Cytokeratin 17 antibody, Abcam, Cat#Ab53707;
Rabbit anti-Cytokeratin 7 antibody, Abcam, Cat#Ab181598;
Mouse anti-GADD 153 Antibody (B-3), Santa Cruz, Cat#sc-7351;
Rabbit anti-GRP78/HSPA5 Antibody, Novus Biologicals, Cat#NB300-520;
Goat anti-Mouse IL-1 beta /IL-1F2 Antibody, R&D Systems, Cat#AF-401-NA;
Rabbit anti-Cyclin D1 antibody [EPR2241], Abcam, Cat#ab134175;
Mouse anti-SQSTM1/p62 antibody, Abcam, Cat#Ab56416;
Mouse anti-Phospho-p70 S6 Kinase (Thr389) (1A5), Cell Signaling, Cat#9206S;
For T cell deletion:
Rat anti-mouse CD8α (clone 2.43), BioXCell, Cat#BE0061;
Rat anti-mouse CD4 (clone GK1.5), BioXCell, Cat#BE0003-1;
Armenian Hamster anti-mouse PD-1 (clone J43), BioXCell, Cat#BE0033-2;
For Flow Cytometry:
TruStain FcX™ (anti-mouse CD16/32) Antibody, Biolegend, Cat#101320;
APC/Cy7 anti-mouse CD45 Antibody, Biolegend, Cat#103116;
Pacific Blue™ anti-mouse CD3 Antibody, Biolegend, Cat#100213;
Alexa Fluor® 647 anti-mouse CD8a Antibody, Biolegend, Cat#100727;
PerCP/Cyanine5.5 anti-mouse CD4 Antibody, Biolegend, Cat#116011;
FITC anti-mouse TCR β chain Antibody, Biolegend, Cat#109205;
PE anti-mouse TCR γ/δ Antibody, Biolegend, Cat#118107;
BV605™ anti-mouse NK-1.1 Antibody, Biolegend, Cat#108740;
BV785™ anti-mouse CD25 Antibody, Biolegend, Cat#102051;
BV421™ anti-mouse/human CD11b Antibody, Biolegend, Cat#101251;
FITC anti-mouse Ly-6G/Ly-6C (Gr-1) Antibody, Biolegend, Cat#108405;
BV650™ anti-mouse F4/80 Antibody, Biolegend, Cat#123149;
BV785™ anti-mouse CD11c Antibody, Biolegend, Cat#117336;
PE anti-mouse/human CD45R/B220 Antibody, Biolegend, Cat#103208;
PerCP/Cyanine5.5 anti-mouse I-A/I-E Antibody, Biolegend, Cat#107625;
PE/Cy7 anti-mouse CD127 (IL-7Rα) Antibody, Biolegend, Cat#135013;
Human/Mouse/Rat FoxP3 Alexa Fluor® 405-conjugated Antibody, R&D Systems, Cat#IC8970V;
PE anti-mouse CD8a Antibody, Biolegend, Cat#100707;
Alexa Fluor® 488 anti-mouse CD4 Antibody, Biolegend, Cat#100425;
PE Hamster Anti-Mouse γδ T-Cell Receptor, BD Biosciences, Cat# 561997;
Mouse IFN-gamma R2 Alexa Fluor® 405-conjugated Antibody, R&D Systems, Cat#FAB773V;
TNF alpha Rat anti-Mouse, PE-eFluor 610, Life Technologies, Cat#LS61732182;
PE/Cy7 anti-mouse IL-17A Antibody, Biolegend, Cat#506921;
APC anti-mouse CD8a Antibody, Biolegend, Cat#100711;
Alexa Fluor® 700 anti-mouse CD45 Antibody, Biolegend, Cat#103127;
APC/Cy7 anti-mouse TCR β chain Antibody, Biolegend, Cat#109219;
PE anti-mouse IL-17A Antibody, Biolegend, Cat#506903;
PE/Cy7 anti-mouse NK-1.1 Antibody, Biolegend, Cat#108713;
BV510™ anti-mouse CD4 Antibody, Biolegend, Cat#100449;
PE anti-mouse CD8a Antibody, Biolegend, Cat#100707;
APC anti-mouse IL-17A Antibody, Biolegend, Cat#506915;
APC/Cy7 anti-mouse Ly-6G Antibody, Biolegend, Cat#127623;
APC anti-mouse Ly-6C Antibody, Biolegend, Cat#128015;

PE/Cy7 anti-mouse CD19 Antibody, Biolegend, Cat#115519;
PerCP/Cy5.5 anti-mouse/human CD45R/B220 Antibody, Biolegend, Cat#103235;
PE-eFluor 610 F4/80 Monoclonal Antibody (BM8), ThermoFisher, Cat#61-4801-82;
PE anti-mouse I-A/I-E Antibody, Biolegend, Cat#107607;
PerCP anti-mouse CD11c Antibody, Biolegend, Cat#117325;
Alexa Fluor® 405 anti-mouse CD11b Antibody (M1/70), Novus Biologicals, Cat#FAB1124V-025;
APC anti-mouse CD8b Antibody, Biolegend, Cat#126613;
PerCP anti-mouse CD25 Antibody, Biolegend, Cat#102027;
BV510™ anti-mouse CD4 Antibody, Biolegend, Cat#100449;
PE-eFluor 610 Ki-67 Monoclonal Antibody, ThermoFisher, Cat#61-5698-82;
PE/Cy7 anti-mouse CD105 Antibody, Biolegend, Cat#120409;
APC anti-GATA3 Antibody, Biolegend, Cat#653805;
BV786 Rat Anti-Mouse IL-17A, BD Biosciences, Cat#564171;
BV605 Rat Anti-Mouse CD119 (IFN-γ), BD Biosciences, Cat#745111;
IL-13 Monoclonal Antibody (eBio13A), PE-Cyanine7, ThermoFisher, Cat#25-7133-82;
BV711 Rat Anti-Mouse TNFa, BD Biosciences, Cat#563944;
Brilliant Violet 421™ anti-human TCR Vα7.2 Antibody, Biolegend, Cat#351715;
BV711 Rat Anti-Mouse CD1d, BD Biosciences, Cat#740711;
FITC-CD44 anti-human mouse antibody, Biolegend, Cat# 103005;
PE/Cy7-CD133 anti-mouse atibody, Biolegend, Cat#141210;
PE/Cy7-CD133 anti-human atibody, Biolegend, Cat#372810;

Validation

For IHC, IF and WB:
Mouse anti-β-Catenin (BDI080), Abcam, Cat#Ab19448; Applications: IP, IHC, IF, ELISA, WB, Reactivity: M, R, S, G, H;
Chicken anti-GFP, Abcam, Cat#Ab13970; Applications: IP, IHC, IF, ELISA, WB, Reactivity: GFP;
Rabbit anti-CD3ε (D4V8L), Cell Signaling, Cat#99940; Applications: IHC, IF, WB, Reactivity: M;
Rabbit anti-CD4 [EPR19514], Abcam, Cat# ab183685; Applications: IHC, IP, IF, WB, Reactivity: M;
Rat anti-CD8a Antibody (4SM15), eBioscience, Cat#14-0808-82; Applications: IHC, FC, IF, Reactivity: M;
Rabbit anti-pZap-70 (65E4), Cell Signaling, Cat#2717; Applications: IHC, FC, WB, Reactivity: H, M;
Mouse anti-mouse I-AK (AαK) (MHC-II) (11-5.2), Biolegend, Cat#110002; Applications: IHC, FC, Reactivity: M;
Mouse anti-mouse H-2Kb/H-2Db, Biolegend, Cat#114602; Applications: IHC, FC, Reactivity: M;
Rabbit anti-Calreticulin Antibody, ThermoFisher, Cat#PA3-900; Applications: IP, IF, IHC, WB, FC, Reactivity: M, R, H;
Mouse anti-ERp46 Antibody (C-11), Santa Cruz, Cat#sc-271667; Applications: IP, IF, IHC, WB, Reactivity: M, R, H;
Rabbit anti-HMGB1 Polyclonal Antibody, ThermoFisher, Cat#PA1-16926; Applications: FC, IF, IHC, WB, Reactivity: M, R, H, S, D, B;
Mouse anti-human LAMP-1 Antibody (H4A3), Biolegend, Cat#328601; Applications: IF, IHC, WB, Reactivity: M, H;
Rabbit anti-eIF2A [pSer51] Antibody, Novus Biologicals, Cat#NB100-81896; Applications: IF, IHC, WB, Reactivity: M, R, H;
Rabbit anti-Phospho-eIF2α (Ser51), Cell Signaling, Cat#9721; Applications: WB, Reactivity: M, R, H;
Rabbit anti-Cleaved Caspase-3 (Asp175) (5A1E), Cell Signaling, Cat#9664S; Applications: IF, IHC, WB, IP, FC, Reactivity: M, R, H;
Mouse anti-Caspase-1 (p20), AdipoGen Life Science, Cat#AG-20B-0042-C100; Applications: IHC, WB, IP, Reactivity: M;
Rabbit anti-PEAR1 Polyclonal Antibody, ThermoFisher, Cat#PA5-21057; Applications: IHC, WB, ICC, Reactivity: M, R, H;
Armenian Hamster anti-CD11c Antibody (AP-MAB0806), Novus Biologicals, Cat#NB110-97871;
Mouse anti-LRP1 Monoclonal Antibody, ThermoFisher, Cat#37-3800; Applications: WB, FC, IF, IHC, Reactivity: H, M.
Mouse anti-KDEL (10C3), Novus Biologicals, Cat#NBP1-97469; Applications: WB, ELISA, EM, FC, ICC/IF, IHC, IP; Reactivity: H, M, R,
Mouse anti-HNF-4-alpha antibody [K9218], Abcam, Cat#ab41898; Applications: IP, IHC, WB, ELISA, ICC, FC, Reactivity: M, R, H;
Rabbit anti-alpha 1 Fetoprotein antibody, Abcam, Cat#ab46799; Applications: IP, IHC, WB, Reactivity: M, R, H;
Rabbit anti-Albumin antibody [EPR20195], Abcam, Cat#ab207327; Applications: IP, IHC, IF, WB, FC, Reactivity: M, R, H;
Mouse anti-HNF-6 Antibody (G-10), Santa Cruz, Cat#sc-376167; Applications: IP, IF, WB, Reactivity: M, H;
Mouse anti-Cytokeratin 19 Antibody (A-3), Santa Cruz, Cat#sc-376126; Applications: IP, IHC, IF, WB, Reactivity: M, R, H;
Mouse anti-Hex antibody, Abcam, Cat#Ab117864; Applications: IHC, IF, WB, Reactivity: M, H;
Rabbit anti-ARF1 Polyclonal Antibody, ThermoFisher, Cat#PA1-127; Applications: IP, IHC, IF, WB, Reactivity: M, D, H;
Mouse anti-GAPDH Antibody (GA1R), ThermoFisher, Cat#MA5-15738; Applications: IP, IHC, IF, WB, ELISA, Reactivity: M, R, H, P;
Mouse Anti-E Cadherin antibody [M168], Abcam, Cat#Ab76055; Applications: IP, IHC, IF, WB, ELISA, FC, Reactivity: M, R, H, H;
Rabbit anti-CEBP Alpha/CEBPA antibody, Abcam, Cat#Ab40764; Application: EMSA, WB, Reactivity: M, R, H.
Rabbit anti-Cytokeratin 17 antibody, Abcam, Cat#Ab53707; Applications: IHC, IF, WB, ELISA, Reactivity: M, R, H;
Rabbit anti-Cytokeratin 7 antibody, Abcam, Cat#Ab181598; Applications: WB, ICC/IF, IHC,
Reactivity: H, M, R;
Mouse anti-GADD 153 Antibody (B-3), Santa Cruz, Cat#sc-7351; Applications: IP, IHC, IF, WB, ELISA, Reactivity: M, R, H;
Rabbit anti-GRP78/HSPA5 Antibody, Novus Biologicals, Cat#NB300-520; Applications: WB, ICC/IF, IHC,
Reactivity: H, M, R;
Goat anti-Mouse IL-1 beta /IL-1F2 Antibody, R&D Systems, Cat#AF-401-NA; Applications: ELISA, WB, Reactivity: M;
Rabbit anti-Cyclin D1 antibody [EPR2241], Abcam, Cat#ab134175; Applications: IP, IHC, IF, WB, Reactivity: M, R, H;
Mouse anti-SQSTM1/p62 antibody, Abcam, Cat#Ab56416; Applications: IP, IHC, IF, WB, FC, Reactivity: M, R, H, M;
Mouse anti-Phospho-p70S6 Kinase (Thr389) (1A5), Cell Signaling, Cat#9206S; Applications: WB, Reactivity: H, M, R, D.
Rabbit anti-CPT1A (8F6AE9), Abcam, Cat#ab128568; Applications: WB, IHC, IF, Flow Cyt, Reactivity: H, M, R.
Rabbit anti-CD36/SR-B3, Novus Biologicals,, Cat#NB400-144; Applications: WB, IHC, IF, Flow Cyt, Reactivity: H, M, R, P, Bv.
Rabbit anti-Fatty Acid Synthase (C20G5), Cell Signaling, Cat#3180T; Applications: WB,IP, IHC,IF, Reactivity: H, M, R.
Mouse anti-Nitrotyrosine, R&D system, Cat#MAB3248; Applications: WB, IF, IHC, Reactivity: H, M, R.
Mouse anti-PD-L1/B7-H11, R&D system, Cat#MAB90781; Applications: WB, IF, IHC, Flow Cyt, Reactivity: M.
For T cell deletion:
Rat anti-mouse CD8α (clone 2.43), BioXCell, Cat#BE0061; Applications: WB, in vivo CD8+ T cell depletion, Reactivity: M.
Rat anti-mouse CD4 (clone GK1.5), BioXCell, Cat#BE0003-1; Applications: WB, in vivo CD4+ T cell depletion, Reactivity: M.
Armenian Hamster anti-mouse PD-1 (clone J43), BioXCell, Cat#BE0033-2; Applications: WB, in vivo blocking of PD-1/PD-L signaling, in vitro PD-1 neutralization, Reactivity: M.

Eukaryotic cell lines

Policy information about cell lines

Cell line source(s)	4T1, ATCC, CRL-2539;<br>B16-F10, ATCC, CRL-6475;<br>A20 [A-20], ATCC, TIB-208;<br>CT26.WT, ATCC, CRL-2638;<br>293T/17 [HEK293T/17], ATCC, CRL-11268;<br>Huh-7, JCRB Cell Bank, JCRB0403;
Authentication	All cell line were authenticated by the company that purchased.
Mycoplasma contamination	All of the cell lines have been tested that negative Mycoplasma contamination.
Commonly misidentified lines<br>(See ICLAC register)	There is no use the misidentified lines.

Animals and other organisms

Policy information about studies involving animals; ARRIVE guidelines recommended for reporting animal research

Laboratory animals	All the mice were used at the age of 6 to 8-week-old, female and male mice, the detailed as follows: Mouse: C57BL/6J, Jackson Laboratories, Cat#000664; BALB/c, Charles River, Cat#028; Arf1f/f mice were generated at NCI-Frederick, NIH; Foxa3-Cre, Mutant Mouse Resources & Research Centers (MMRRC), Cat#011121-MU; B6.Cg-Speer6-ps1Tg(Alb-cre)21Mgn/J (Alb-Cre), Jackson Lab, Cat#003574; SJL-Tg(ACTFLPe)9205Dym/J (Actb-Flp1), Jackson Lab, Cat#005703; B6.129P2-Lgr5tm1(cre/ERT2) Cle/J (Lgr5-creER), Jackson Lab, Cat#008875; C57BL/6-Apctm1Tyj/J (Apcf/f), Jackson Lab, Cat#009045; B6.Cg-Tg(Cebpb-tTA)5Bjd/J (Cebpb-tTA), Jackson Lab, Cat#003563; FVB/N-Tg(tetO-MYC)36aBop/J (TetO-MYC), Jackson Lab, Cat#019376; B6.129(Cg)-Axin2tm1(cre/ERT2)Rnu/J (Axin2-CreER), Jackson Lab, Cat#018867; B6.129S7-Rag1tm1Mom/J (Rag1-KO), Jackson Lab, Cat#002216; C.129S7(B6)-Ifngtm1Ts/J (IFNg-KO), Jackson Lab, Cat#002286; NU/J, Jackson Lab, Cat#002019; B6.129P2-P2rx7tm1Gab/J (P2X(7)R-KO), Jackson Lab, Cat#005576, B6.FVB-1700016L21RikTg(Itgax-DTR/EGFP)57Lan/J (CD11c-DTR), Jackson Lab, Cat#004509, B6.129S4-Gt(ROSA)26Sortm1Sor/J (R26R), Jackson Lab, Cat#003474. For CreER induction, mice were i.p. injected with tamoxifen (100mg/kg mice) in corn oil for 3 to 5 consecutive days.
Wild animals	The study did not involve wild animals.
Field-collected samples	The study did not involve samples collected from the field.
Ethics oversight	All breeding and experimental procedures were performed in accordance with the ACUC guidelines and regulations and with the approval of the ACUC at NCI-Frederick, NIH.

Note that full information on the approval of the study protocol must also be provided in the manuscript.

Flow Cytometry

Plots

Confirm that:

☒ The axis labels state the marker and fluorochrome used (e.g. CD4-FITC).

☒ The axis scales are clearly visible. Include numbers along axes only for bottom left plot of group (a 'group' is an analysis of identical markers).

☒ All plots are contour plots with outliers or pseudocolor plots.

☒ A numerical value for number of cells or percentage (with statistics) is provided.

Methodology

Sample preparation	Mouse intestine IEL and LPL immune cells were isolated as follows, remove the colon and small intestine from mouse and clear small intestine (SI) of fecal matter. All the peyers patches were cut off from small intestine after clear the fecal matter. The small intestine/colon was washed in ice cold RPMI1640 medium for 3 times 5 min. The SI was cut into 1-3 cm pieces in length and put in a 50 ml beaker with 10 ml of pre-warmed IEL isolation media. The SI pieces were put into the flask with a stir bar, covered with aluminum foil and incubated for 20 minutes at 37°C with stirring. The contents of the flask were pass through a sterile 200 µm kitchen strainer into a 100 ml beaker on ice. The pieces of the SI were transferred to a 50 ml tube and added 10 ml of shake media (RMPI1640, 100 units/ml penicillin/Streptomycin, and 2 mM EDTA). Shake the tube vigorously for 30 seconds and transferred the contents of the tube through a 200 µm kitchen strainer into the same beaker. Repeat twice of the above procedure. The beaker on ice is the IEL.<br>For isolation of IEL, the solution in the beaker was filtered by a 70 µm cell strainer into a 50 ml tube and spin down at 1200 rpm for 7minutes at 4°C. Re-suspend the pellet in RPMI1640 + 3% FBS and pass through a 40 µm cell strainer. Spin down again and resuspend the pellet in 40 ml of 30 % Percoll and the cells were centrifuged for 20 minutes with no brake at 1600 rpm. After centrifuge, the supernatant was carefully removed using a pipette. Resuspend the cell pellet and transfer to a clean tube. Wash the cell pellet at least once before staining.

For isolation of LPL from SI, the pieces of SI were put into a 50 ml beaker contain 10 ml of RMPI1640, 100 units/ml penicillin/ Streptomycin, liberase (I:250) and 0.05% DNase. Mince gut pieces by scissors. Add a stir bar and incubate for 30 minutes at 37°C with stirring. After incubating, pour solution through a 70 µm cell strainer into a 50 ml tube. Mash the remaining pieces of gut through the filter. Wash the beaker with RMPI1640 + 3% BSA and pass this through the filter again. Centrifuge the cell suspension for 7 minutes at 1200 rpm. Resuspend pellet and pass through a 40 µm filter. Spin down the cells and resuspend the cell pellet in 30 % percoll 1 x PBS. Mix well and centrifuged for 10 minutes at 1200 rpm in 4°C. The pellet is LPL immune cells ready for stain and FACS.
Mice liver immune cells were isolated by dissection of the entire live and placed into a 60 mm peri dish. The liver was minced to small parts and homogenized with a syringe plunger against the 70 µm mesh screen. Collection of the pass-through cells to filter again with a 70 µm strainer into a 50 ml tube. The single cell suspension was added into 33% percoll and mix well for centrifuge 20 minutes at 2000 rpm in room temperature. The pellet is red blood cell s and lymphocytes. Wash the pellet with RBC lysis buffer (Biolegend, Cat#420301) for 3 minutes, and the cell supernatants were added RPMI medium for spin 5 minutes at 1500 rpm at 4°C. After that, the cells pellet was ready to stain for flow cytometry.

Instrument

BD Fortessa SORP

Software

BD Fortessa SORP was operated using BD FACSDiva Software to collect the FACS data.
FlowJo software was used to analysis the FACS plots.

Cell population abundance

This study only analysis the immune cells number in the intestine and liver, WBC gating and acquisition were based on using splenocytes to set the gates for the different WBC populations, and WBC numbers from associated liver or intestine tissue were variable due to the variability of WBC infiltration of those tissues within the experimental group. Numbers of Live, CD45+ cells from liver ranged from 100 events (no infiltration) up to 27,000 events (higher infiltration).

Gating strategy

Cells were gated on FSC x SSC to exclude debris, then FSC-A x FSC-H to exclude doublets, gated for “live” cells based on LIVE/ DEAD Fixable Aqua exclusion, then gated on CD45-Positive cells (definitively CD45 Positive based on unstained controls and clear, bimodal expression of CD45), then individual WBC gates defined based on WT negative control and clear bimodal staining of WBC populations based on splenocytes used as positive controls.

☒ Tick this box to confirm that a figure exemplifying the gating strategy is provided in the Supplementary Information.
